# Consensus recommendation for prenatal, neonatal and postnatal management of congenital cytomegalovirus infection from the European congenital infection initiative (ECCI)

**DOI:** 10.1016/j.lanepe.2024.100892

**Published:** 2024-04-01

**Authors:** Marianne Leruez-Ville, Christos Chatzakis, Daniele Lilleri, Daniel Blazquez-Gamero, Ana Alarcon, Nicolas Bourgon, Ina Foulon, Jacques Fourgeaud, Anna Gonce, Christine E. Jones, Paul Klapper, André Krom, Tiziana Lazzarotto, Hermione Lyall, Paulo Paixao, Vassiliki Papaevangelou, Elisabeth Puchhammer, George Sourvinos, Pamela Vallely, Yves Ville, Ann Vossen

**Affiliations:** a Université Paris Cité, URP 7328 FETUS, F-75015, Paris, France; b Virology Laboratory, Reference Laboratory for Cytomegalovirus Infections, Hôpital Necker Enfants Malades, GHU Paris Centre, AP-HP, Paris, France; c Obstetrics, Fetal Medicine Surgery and Imaging Unit, Hôpital Necker Enfants Malades, GHU Paris Centre, AP-HP, Paris, France; d Second Department of Obstetrics and Gynecology of Aristotle University of Thessaloniki, Thessaloniki, Greece; e Microbiology and Virology Unit, Fondazione IRCCS Policlinico San Matteo, Pavia, Italy; f Pediatric Infectious Diseases Unit, Hospital Universitario 12 de Octubre, Instituto de Investigación Hospital 12 de Octubre (imas12), Universidad Complutense, Madrid, Spain; g Department of Neonatology, Hospital Hospital Sant Joan de Déu, BCNatal (Barcelona Center for Maternal, Fetal and Neonatal Medicine), Institut de Recerca Sant Joan de Déu, Universitat de Barcelona, Barcelona, Spain; h Department of Otorhinolaryngology and Head & Neck Surgery, Vrije Universiteit Brussels, University Hospital UZ Brussel, Brussels Health Campus. De Poolster, Rehabilitation Centre, Brussels, Belgium; i BCNatal: Fetal Medicine Research Center (Hospital Clínic and Hospital Sant Joan de Déu), Universitat de Barcelona, Barcelona, Spain; j Faculty of Medicine and Institute for Life Sciences, University of Southampton and NIHR Southampton Clinical Research Facility and NIHR Southampton Biomedical Research Centre, University Hospital Southampton NHS Foundation Trust, United Kingdom; k Microbiology and Virology Unit (EIGen), School of Biological Sciences, University of Manchester, Manchester, M139PT, UK; l Department of Medical Ethics and Health Law, Leiden University Medical Center, Leiden, the Netherlands; m Microbiology Unit, IRCCS Azienda Ospedaliero-Universitaria di Bologna, Bologna, Italy; n Department of Medical and Surgical Sciences (DIMEC), University of Bologna, Bologna, Italy; o Imperial College Healthcare NHS Trust, London, England; p CHRC, NOVA Medical School/Faculdade de Ciências Médicas, Universidade NOVA de Lisboa, 1169-056, Lisbon, Portugal; q Third Department of Pediatrics, School of Medicine, National and Kapodistrian University of Athens, Greece; r Center for Virology, Medical University Vienna, Austria; s Laboratory of Clinical Virology, Medical School, University of Crete, Heraklion, Crete, 71003, Greece; t Department of Medical Microbiology, Leiden University Medical Center, Leiden, the Netherlands

**Keywords:** Congenital cytomegalovirus infection, Guidelines, Prenatal, Neonatal, Postnatal

## Abstract

Congenital cytomegalovirus (cCMV) infection carries a significant burden with a 0.64% global prevalence and a 17–20% chance of serious long-term effects in children. Since the last guidelines, our understanding, particularly regarding primary maternal infections, has improved. A cCMV guidelines group was convened under the patronage of the European Society of Clinical Virology in April 2023 to refine these insights. The quality and validity of selected studies were assessed for potential biases and the GRADE framework was employed to evaluate quality of evidence across key domains. The resulting recommendations address managing cCMV, spanning prevention to postnatal care. Emphasizing early and accurate maternal diagnosis through serological tests enhances risk management and prevention strategies, including using valaciclovir to prevent vertical transmission. The guidelines also strive to refine personalized postnatal care based on risk assessments, ensuring targeted interventions for affected families.

**Key messages**

• Maternal CMV serology should be performed in the first trimester of pregnancy, as cCMV sequelae are limited to maternal infection acquired in the first trimester of pregnancy.
• In cases of maternal primary infection in the periconceptional period or in the first trimester, oral valaciclovir at a dose of 8 g/day should be administered as early as possible after the diagnosis and until the amniocentesis.
• A negative CMV PCR in amniotic fluid following timely amniocentesis ensures absence of long-term sequelae.
• Newborns with CNS-related symptoms but also those with isolated SNHL, should be treated with valganciclovir. Treatment should be started as soon as possible and before 1 month of age, however treatment initiated between 1 and 3 months may also be beneficial.
• Children with cCMV and confirmed transmission in the first trimester or unknown timing of transmission should be followed up to at least 6 years of age to ensure specialized management. For those with documented maternal primary infection in the second and third trimesters of pregnancy this follow-up may not be necessary

## Introduction

The burden of congenital cytomegalovirus (cCMV) infection is high, with a global prevalence of 0.64%^1^ and a 17–20% risk of permanent sequelae in infected children.^2^ Expert consensus guidelines for its diagnosis and management were published in 2017.^3^^,^^4^ However, since then two major advances have been made concerning cCMV following a primary maternal infection. Firstly, demonstration of the efficacy of antiviral treatment in preventing vertical transmission in pregnant women with a primary infection^5^^,^^6^; secondly, evidence indicating that the risk of major sequelae is limited to maternal infection in the first trimester of pregnancy.^7^ Against this backdrop, a transdisciplinary group of experts in cCMV (European Congenital Cytomegalovirus Initiative; ECCI) (ecci.group) met to examine and classify the available data (Table 1, Table 2, Table 3, Table 4).

**Table 1:** Recommendations on primary prevention, awareness and diagnosis of maternal infection.

Recommendation
We recommend advising women on hygienic measures prior to pregnancy (or as soon as possible once pregnant) especially those known CMV seronegative. **Grade B**
We recommend implementing strategies to improve the education of women of childbearing age. **Grade C**
We recommend improving knowledge among healthcare professionals caring for pregnant women/childbearing age women. **Grade B**
We recommend an EU uniform policy for prevention of primary CMV infection in pregnancy. **Grade D**
We recommend that women with equivocal CMV IgG results should be considered as seronegative. **Grade D**
We recommend to perform CMV serology in the first trimester of pregnancy as early as possible followed in seronegative women by a retest every 4 weeks until 14–16 weeks. CMV serology is not recommended in pregnant women beyond 16 weeks except in cases with ultrasound CMV compatible symptoms **Grade A**
Consideration depending on local CMV epidemiology should be given to CMV serology universal screening in the first trimester in women with unknown CMV serostatus or known as seronegative. **Grade D**
We recommend to use IgG and IgM testing to diagnose a maternal primary infection. **Grade B**
We recommend using IgG avidity testing to exclude a recent (less than 90 days) maternal primary infection in cases with positive IgM and positive IgG. **Grade B**
We recommend using a second avidity test for sera with positive IgM, positive IgG and intermediate IgG avidity value. **Grade D**
We do not recommend testing for CMV PCR in blood or in urine since it is not helpful for dating maternal primary infection in women with positive IgG and IgM. **Grade B**
In cases with an isolated positive IgM, a CMV PCR test in whole blood may exclude if negative or confirm if positive an ongoing primary infection. **Grade D**
We do not recommend testing CMV serology or CMV PCR in blood or urine in women known to be seropositive before pregnancy. **Grade B**

**Table 2:** Recommendations on secondary prevention, diagnosis of fetal infection and follow-up of infected fetuses.

Recommendation
We recommend the administration of oral valacyclovir at a dose of 8 g/day in cases with maternal primary infection in the periconceptional period or the first trimester of pregnancy, as early as possible after the diagnosis and until the result of the CMV PCR in amniocentesis. **Grade A**
We recommend the dose regiment of 2 g 4 times per day to minimize the risk of renal side effects. **Grade D**
We recommend against the administration of hyperimmune globulin, at doses of 100 IU/kg every 4 weeks, in pregnant women with primary CMV infection. **Grade A**
Administration of hyperimmune globulin at dose of 200 IU/kg every 2 weeks, in women with very recent primary CMV infection in the first trimester may be considered. **Grade C**
We recommend performing CMV PCR in amniotic fluid collected from 17 + 0 weeks gestation for the diagnosis of fetal CMV infection, provided that maternal infection occurred at least 8 weeks earlier. **Grade B**
Fetal ultrasound assessment and MRI assessment in the third trimester is recommended in infected fetuses, as it can provide information regarding the presence of CMV associated findings which will provide prognostic information. **Grade A**
In women with confirmed fetal infection, fetal treatment with valacyclovir 8 g/day may be considered after discussion with an expert team. **Grade C**
We recommend reassurance in women with negative CMV PCR in amniotic fluid since late fetal infection (after the amniocentesis) is not associated with long term sequelae. **Grade A**

**Table 3:** Recommendation for neonatal diagnosis.

Recommendation
We recommend performing a CMV PCR on a sample collected in the first three weeks of life to confirm a diagnosis of cCMV. **Grade D**
We recommend using CMV PCR on urine or saliva for the diagnosis of cCMV in neonates. A positive CMV PCR on saliva should be confirmed by a CMV PCR on a urine sample. **Grade B**
We recommend performing CMV PCR on DBS for retrospective diagnosis of cCMV infection, keeping in mind that the negative predictive value of this test depends on the sensitivity of the assay. **Grade A**
We recommend not performing IgM CMV testing in the neonate for cCMV diagnosis. **Grade B**
We recommend testing for cCMV in all infants born to mothers with suspected or confirmed primary CMV infection during pregnancy. **Grade A**
We recommend testing for cCMV in infants with abnormalities on fetal imaging potentially associated with CMV. **Grade D**
We recommend testing for cCMV in any infant with suspected hearing loss at birth. **Grade A**
We recommend testing for cCMV in infants with symmetric IUGR (i.e., weight and head circumference both affected) of unknown etiology. **Grade D**
We recommend testing very preterm infants (<32 weeks gestational age) or very low birth weight infants (<1500 g) at birth, in order to differentiate between congenital and postnatal CMV infection. **Grade D**
We recommend testing for CMV in infants with unexplained symptoms, laboratory abnormalities and/or image findings consistent with cCMV. **Grade D**

**Table 4:** Recommendation on neonatal investigation, neonatal treatment and follow-up.

Recommendation
In cases of unknown type and timing of maternal infection, we recommend performing retrospective maternal serology on stored samples, when available, to determine the type and timing of maternal infection. **Grade A**
As the risk of long-term sequelae is similar, we recommend the same investigation of neonates with cCMV, whether maternal infection was primary, non-primary, or unknown. **Grade A**
We recommend a complete anthropometric and physical examination at birth. **Grade A**
We recommend performing full blood count, liver enzymes and bilirubin (total and conjugated) at birth. **Grade A**
We recommend ophthalmologic assessment at birth. **Grade A**
We recommend audiologic assessment at birth. **Grade A**
We recommend performing an MRI in all infants with clinical manifestations at birth, SNHL, chorioretinitis or abnormalities detected on cUS. MRI could be undertaken in cases of known maternal CMV primary infection during the first trimester, or where timing of transmission is not known. **Grade B**
We recommend the use of fetal and neonatal neuroimaging scoring systems to evaluate the individual risk of long-term sequelae. **Grade C**
We recommend against lumbar puncture for the diagnosis or assessment of cCMV, even in infants with symptomatic infection. **Grade B**
We recommend 6 months of antiviral treatment in newborns with significant CMV-related symptoms at birth. **Grade B**
We recommend antiviral treatment in infants with cCMV and isolated hearing loss. **Grade C**
We recommend valganciclovir as the treatment of choice. Ganciclovir may be used for infants unable to take enteral medication or in very severe cases, switching to oral route as soon as possible. **Grade B**
We recommend checking the full blood count and liver function tests regularly during antiviral treatment. **Grade B**
We recommend starting antiviral treatment as soon as possible, and before 1 month of age. **Grade A**. Treatment initiated between 1 and 3 months may have benefit. After 3 months of age, case-by-case discussion with an expert is recommended. **Grade C**
We recommend 6 weeks of antiviral treatment in infants with isolated persistent hepatitis and no other manifestations of cCMV at birth. **Grade D**
We recommend 6 weeks of antiviral treatment in infants with isolated persistent thrombocytopenia and no other manifestations of cCMV at birth. **Grade D**
We do not recommend treatment of infants with isolated IUGR, without other manifestations of cCMV at birth. **Grade D**
Ophthalmological follow-up is only recommended for those infants with retinitis at birth and not required for newborns with normal retinal examination. **Grade B**
We recommend that children with cCMV and confirmed transmission in the first trimester or unknown timing of transmission should be follow-up from birth, through treatment, at 6, and 12 months of age, then annually to school age (by Pediatric Infectious Diseases or General Pediatrics). **Grade D**
We recommend that children with clinical symptoms at birth and/or evidence of long-term sequelae (neurologic disease, SNHL, chorioretinitis and/or neurodevelopmental impairment) should be seen on an annual basis at least up to 6 years of age to ensure specialized management. **Grade A**
We recommend neurodevelopmental assessment at 24–36 months of age in high-risk children, and further follow-up and interventions according to findings. **Grade D**
We recommend evaluation by a pediatric neurologist in all children with neurological symptoms and/or significant findings on neuroimaging as well as in children with neurological concerns that arise during follow-up. **Grade A**
Asymptomatic children with normal imaging and documented maternal primary infection in the second or third trimester may follow standard pediatric care. **Grade A**
In infants with normal hearing at birth, with unknown timing of CMV infection during pregnancy, or known first trimester infection, we recommend hearing follow-up until at least 5 years of age. **Grade A**
The estimated risk of delayed SNHL for asymptomatic children without SNHL at birth and with a proven MPI in the second trimester is low, and there is no full consensus on whether these cases need hearing follow-up. **Grade D**
In children with a proven MPI in the third trimester and normal hearing at birth, we do not recommend hearing follow-up. **Grade A**
In cases of hearing loss at birth, we recommend regular hearing testing for as long as required (can be lifelong). **Grade A**
Vestibular screening tests should be performed within the first year of life in high-risk children (those with first trimester maternal infection, with unknown timing of maternal infection, with hearing loss or developmental delay). **Grade B**

## Methods

Clinicians specialized in the diagnosis, prognosis, prevention and treatment of cCMV from eight European countries were invited to a workshop organized by the European Society of Clinical Virology (ESCV) in April 2023. The group representing ECCI formulated the questions to be addressed (supplementary) and assessed them prior to the workshop through a systematic and comprehensive literature search of relevant databases including PubMed, Scopus, Cochrane library and sources of grey literature, using the search terms “Congenital Cytomegalovirus Infection”, “Congenital CMV”, “pregnancy”, “prenatal”, “neonatal”, “postnatal” and “long-term” until September 2023. Only papers published in English were reviewed. The final reference list was generated on the basis of originality and relevance to the broad scope of this Review. Identified studies were thoroughly reviewed and relevant data extracted.

The quality and validity of the selected studies were assessed for potential bias using the Cochrane Risk of Bias tool for randomized controlled trials (RCTs), the ROBINS-I tool for non-randomized interventional studies, the Newcastle Ottawa Scale for cohort, case–control and cross-sectional studies, and the Quality Assessment of Diagnostic Accuracy Studies-2 tool for diagnostic studies.^8^, ^9^, ^10^, ^11^ Risk of bias was assessed in terms of selection, performance, detection, attrition, reporting bias, outcome assessment and duration of follow-up.

The GRADE framework was used to assess the quality of evidence in key areas. Initial quality scores were assigned on the basis of study design, with RCTs generally considered high-quality evidence, as were high-quality meta-analyses and systematic reviews. Quality scores were adjusted based on assessment of risk of bias, inconsistency, indirectness and potential publication bias. This nuanced assessment provided a transparent and standardized evaluation of the quality of evidence.^12^^,^^13^

### Role of funding source

European Society for Clinical Virology (ESCV), ESCV financed the ECCI expert workshop in April 2023.

### Ethics committee approval

Not applicable.

## Evidence and recommendation

### Primary prevention

cCMV can occur after maternal primary infection (MPI) or non-primary maternal infection (NMPI) (Table 1). The incidence of MPI is 1–2% with a vertical transmission rate of 32%.^1^^,^^14^ Epidemiology of MNPI is poorly documented, a meta-analysis reported CMV shedding in 21.5% (95% CI, 12.7%, 30.3%) of seropositive pregnant women, and with a vertical transmission rate probably low <3.5%.^15^^,^^16^ In Europe, with a seroprevalence of 50–85% in pregnant women, around half of cCMV occur after a MPI.^17^

### Primary prevention with hygienic preventive measures

#### In seronegative women prior to and in early pregnancy

Exposure to young children is the main risk factor for MPI, as infected children excrete the virus in their urine and saliva over a long period. Several studies have suggested that hygiene measures considerably reduce the risk of contracting a MPI during pregnancy.^18^, ^19^, ^20^ There is little data on the role of sexual exposure as a cause of MPI.^21^

The highest risk of MPI is for young, parous women born in high-resource countries and who conceive another pregnancy within two years.^22^

#### In seropositive women prior to and in early pregnancy

No clear risk factors for MNPI or cCMV have been identified. In particular, exposure to toddlers has not been associated with MNPI^16^^,^^23^ or cCMV in Europe and Japan,^17^^,^^24^^,^^25^ contrary to what has been observed in Brazil.^26^ At present, there is no proof of the benefit or lack of benefit of hygiene measures in preventing MNPI.

Whilst vaccine candidates aiming to reduce the risk of cCMV are progressing through clinical trials, is it likely to be several years before a licensed product is available, therefore modification of hygiene-based behaviors are the only strategy for primary prevention available.

Despite the lack of data in seropositive women, we recommend advising on hygienic modifications for all women whatever their CMV serostatus. The effectiveness of this prevention depends on starting the measures prior to conception and throughout the first trimester in order to reduce the risk of cCMV-related disabilities.

### Strategies for primary prevention

In Europe, the general population has little knowledge of CMV.^27^ Specifically, only 20–40% of pregnant women have heard of CMV among which 10–15% are aware of preventive measures.^28^ Moreover, gaps in the knowledge of perinatal healthcare professionals have been identified.^28^^,^^29^ There is a need to improve education strategies to inform women about CMV and CMV prevention, ideally before pregnancy and during the first trimester of pregnancy. There is no uniform EU policy on the prevention of CMV infection during pregnancy. A number of recent educational interventions (films, brochures, calendar reminders) have improved hygiene measures and are highly acceptable to pregnant women.^30^^,^^31^

### Diagnosis of maternal infection

Most European countries do not do routine serology in pregnancy and the relevance of this screening needs to be evaluated in each country, based on local epidemiology and cost-effectiveness (Table 1). For example, a recent French cost-effectiveness study showed that universal screening in conjunction with valaciclovir treatment would be cost-effective compare to current practice.^32^

### Establishing CMV serological status

CMV serological status is essential to identify women at risk of MPI during pregnancy. Latest generation CMV IgG assays have high sensitivity (97–100%) and specificity (96–100%).^14^ Discordant CMV IgG results are reported for 1.0–2.6% of samples, mainly those with IgG levels below twice the cut-off value of the test.^32^ We recommend repeating weakly positive IgG results with a second assay or sending the sera to a reference laboratory. Sera that are positive with both assays can be declared positive; those with discordant results should be considered equivocal and declared negative.

### Timing of CMV serology in pregnancy

A meta-analysis reported that the risk of long-term sequelae in a fetus with MPI in the first trimester is 23% (95% CI, 15.4–30.2), compared with 0.1% (95% CI, 0–0.8) and 0% (95% CI, 0–2.1) after MPI in the second or third trimester respectively.^7^ Moreover, in the light of the recent developments regarding the effect of valaciclovir in the prevention of vertical transmission, we recommend an initial serology as soon as possible, followed in seronegative women by a retest every 4 weeks until 14–16 weeks.

Serology is also indicated in pregnant women with symptoms compatible with primary CMV infection, such as prolonged moderate fever, mononucleosis syndrome or elevated liver transaminases. CMV serology may also be done when abnormal ultrasound features suggest fetal infection. In these cases, a negative serology excludes fetal infection and a serology with positive IgG, regardless of the value of IgM or IgG avidity, cannot exclude fetal infection.

### Diagnosis of a CMV primary infection

#### IgG and IgM testing

The diagnosis of MPI is based on CMV IgM detection. The latest generation IgM assays have a high sensitivity (>98%) for detecting MPI in the previous month.^33^, ^34^, ^35^, ^36^, ^37^, ^38^ IgM detection decreases with time, and depending on the assay, IgM detection in the second and third months after MPI ranges from 86 to 97% and 51–90% respectively.^33^^,^^38^ Performance characteristics of IgM assays need to be taken into account (Figure S1).

CMV IgM testing has a poor specificity for diagnosing a recent MPI. CMV IgM is present in 50–80% of sera for up to 6 months after MPI^33^^,^^38^ and cross-reactivity as well as non-specific reactivity may occur. Routine screening studies have reported positive IgM in 0.9–5.7% of women in their first trimester, with a positive predictive value of 16.4% for recent MPI (Table S1). It is therefore essential to perform an IgG avidity test in sera with positive IgM, to exclude or confirm a MPI.

#### IgG avidity testing

Latest generation IgG avidity assays allow excluding a recent MPI (<3 months) and a semi-recent MPI (>3 months/<6 months) with a high sensitivity (94–100% and 80–90% respectively).^34^^,^^36^^,^^38^, ^39^, ^40^ Therefore, high IgG avidity in the first trimester allows with a high probability to exclude a MPI in the first trimester, in the periconceptional (±2 weeks from conception) and in the preconceptional (2–8 weeks before conception) periods. Knowing that the respective vertical transmission rate in these 3 periods has been estimated at 36.8%, 21% and 5.5%.^7^

In recent MPI, a low or an intermediate avidity is found in 85–90% and in 10–15% of sera respectively. In semi-recent MPI avidity is low, intermediate or high in 10–50%, 30–70% and 10–20% respectively. Six months after MPI, IgGs with intermediate avidity are still found in 20–40% of sera.^39^^,^^40^ We recommend that sera with an intermediate avidity result be retested with another avidity assay or be sent to an expert laboratory. If the second assay produces a high avidity value, recent MPI is unlikely. Pregnant women in their first trimester with positive IgM together with low or confirmed intermediate IgG avidity are eligible for secondary prevention.

#### CMV PCR in blood and urine

Sensitivity of CMV PCR in whole blood for the diagnosis of MPI is 100% and 88–97% in the first 2 and 4 weeks respectively (Table S2). The specificity of a positive blood PCR for the diagnosis of recent MPI is low since DNAemia decreases over time to reach negativity after one year but with large individual differences. A positive CMV PCR in urine was reported in 90%, 95%, 70%, 45% and 12% of MPI occurred 1, 2, 6, 12 and 24 months before (Table S2). Therefore, PCR in blood or urine are not reliable to determine the timing of MPI. Exceptions to this rule include cases with isolated positive IgM to CMV, where PCR testing in whole blood could be helpful to exclude or confirm an ongoing MPI.

### CMV diagnosis in pregnant women with preexisting immunity

There is no valid laboratory test to identify women with preexisting immunity at risk of giving birth to an infected neonate. CMV serology and PCR are not helpful with a 0–25% IgM detection rate, a rise in IgG titers in 0–22%, and a positive DNAemia reported in 24–66% of these women (Table S3).

## Secondary prevention, diagnosis of fetal infection and follow-up of infected fetuses

### Secondary prevention

#### Prevention of fetal infection by valaciclovir

In 2020, a RCT assessed the effect of oral valaciclovir at a dose of 8 g/day and showed a 71% reduction in vertical CMV transmission in women with PI acquired periconceptionally or during the first trimester of pregnancy (Table 2).^5^ This RCT was followed by two quasi-randomized trials showing similar results.^41^^,^^42^ A meta-analysis of individual data in the aforementioned studies, showed that administration of oral valaciclovir at a dose of 8 g/day reduces vertical transmission of CMV by 70%, in PI acquired periconceptionally and in the first trimester.^6^ Moreover, the probability of vertical transmission increased with gestational age at the beginning of treatment. This finding indicates that the earlier treatment starts the more effective it is. Therefore, we recommend the administration of oral valaciclovir at a dose of 8 g/day in cases with MPI in the periconceptional period and the first trimester of pregnancy, started as soon as possible after infection until the time of amniocentesis. Earliest possible initiation of treatment is crucial. In order to achieve this, CMV serology needs to be performed as early as possible in the first trimester with a retest between 14 and 16 weeks in seronegative women. In the meta-analysis, mild side effects (nausea or headache) were reported by 21% of women and mild to moderate acute renal failure, which resolved after cessation of treatment, was reported in 3 cases (2%): one after a 2gx4/day regimen^41^ and two after a 4gx2/day regimen.^6^ One study reported acute renal failure in 4% (2/50) of women treated with the 4gx2/day compared to 0 (0/173) women treated with 2gx4/day suggesting that a 2gx4/day regimen should preferably be recommended^43^ (Table S4).

#### Prevention of fetal infection by hyperimmune globulin (HIG)

Two RCTs concluded that intravenous administration of 100 IU/kg HIG every 4 weeks was not effective in preventing vertical transmission of CMV during the first and second trimester.^44^^,^^45^ Therefore, we recommend against this secondary prevention strategy.

A case–control study used HIG 200 IU/kg every 2 weeks in pregnant women with very recent MPI in the first trimester with a 70% reduction in vertical transmission rates.^46^ Thus, the earliest possible administration of HIG at dose of 200 IU/kg every 2 weeks may be considered.

### Diagnosis of fetal infection and follow-up of infected fetuses

#### Diagnosis of fetal infection

CMV PCR on amniotic fluid (AF) is the gold standard for diagnosing fetal CMV infection. Based on early studies, most guidelines recommend performing the PCR on AF collected at or after 21 weeks' and at least 6 weeks after MPI for optimal sensitivity (Table S5). However, a recent study reporting the performance of CMV PCR in 2706 cases showed that PCR on AF is a reliable method to diagnose fetal infection from 17 weeks' onwards, provided that amniocentesis is performed at least 8 weeks after MPI.^47^ In these latter conditions, the specificity of CMV PCR on AF is close to 100% and the sensitivity is around 87–95%. In a meta-analysis, the 8% of neonates found to be infected after a timely negative amniocentesis had no sequelae at 2–3 years’ follow-up.^48^ A possible explanation for these cases is a delayed vertical transmission followed by a late fetal infection (after the first trimester) with therefore no clinically relevant consequences.

#### Follow-up of the pregnancy after fetal diagnosis

Women with a negative amniocentesis should benefit usual antenatal care and preventive therapy should be discontinued.^48^ Women with confirmed fetal infection should benefit from serial focused fetal ultrasound assessment as well as magnetic resonance imaging (MRI) in the third trimester, as it provides complementary information relevant to the prognosis.^49^ Neuroimaging findings are classified into severe and mild (Table S6), and extracerebral findings can also be observed.^50^ The negative predictive value of normal ultrasound and MRI for moderate to severe sequelae is close to 100% with a 17% residual risk of unilateral sensorineural hearing loss (SNHL).^49^^,^^50^ Severe cerebral abnormalities are associated with a poor prognosis. Isolated extracerebral features carry a 30% risk of sequelae. In women who refuse amniocentesis, we recommend discontinuation of valaciclovir preventive therapy between 17 and 18 weeks and proposing serial fetal ultrasound assessment follow-up A case–control study suggested a benefit of 2gx4/day valaciclovir treatment from amniocentesis until birth, with a decreased proportion of symptomatic neonates from 66% without treatment to 18% with treatment and no noticeable maternal or fetal side effects.^14^^,^^51^ Cases of valganciclovir antenatal treatment have been reported, however this is not recommended at this point in time.^14^ In proven fetal infection, contact with an expert team to discuss antenatal treatment is recommended.

## Neonatal diagnosis

### Diagnosis of neonatal infection

#### Timing of neonatal diagnosis

In order to distinguish congenital from postnatal infection, PCR should be performed on a sample collected within 3 weeks of birth, ideally as soon as possible after birth (Table 3).

#### Urine or saliva

Saliva collection has a high acceptability among parents and is easier to collect than urine. The comparison of CMV PCR on saliva versus urine (Table S7) shows high sensitivity (93–100%) and predictive negative value (98–99%) and moderate specificity (91–99.7%) and low positive predictive value (49–73%). False positive results in saliva may be due to contamination from the genital tract or recent breast-feeding and generally have a low viral load. A positive CMV PCR results on a saliva sample should be confirmed with a CMV PCR on a urine sample.

#### Dried blood spots (DBS)

DBS routinely collected in the first week after birth can be tested retrospectively to detect CMV DNA in neonatal blood by PCR to allow a diagnosis in children older than three weeks of age. The sensitivity of DBS testing for diagnosis of cCMV is debated because CMV viral load in the blood of neonates is significantly lower than that in saliva or urine and may even be undetectable, and also due to the different methodologies used for CMV PCR testing in different laboratories. A meta-analysis showed a pooled sensitivity and specificity of 84.4%–99.9%.^52^ CMV PCR on DBS is the gold standard for retrospective diagnosis of cCMV, but may miss cases.

#### Maternal or neonatal serology

The sensitivity of IgM testing in the neonate is low and therefore not recommended for neonatal diagnosis of cCMV.^53^^,^^54^ A negative CMV IgG test at birth in mother or neonate rules out cCMV, however a positive CMV IgG test at birth cannot confirm or exclude cCMV and therefore CMV serology is not recommended for screening or diagnosis in the neonatal period.

### Indications for testing for cCMV infection at birth

Evidence of MPI during pregnancy. With an overall 32% risk of vertical transmission, this population has a high risk of cCMV.^1^

Presence of indicative features on prenatal ultrasonography or MRI (Table S6).

Neonatal clinical manifestations consistent with cCMV (Table S8) including petechiae, hepatosplenomegaly, jaundice, microcephaly and thrombocytopenia. Milder or isolated clinical or laboratory features are not specific to newborns with cCMV and it is more difficult to list which of them warrant testing for cCMV. The most compelling evidence exists for SNHL either bilateral or unilateral.^55^ Routine CMV testing in intrauterine growth restriction (IUGR) or preterm infants has low diagnostic yield (Table S9). We suggest that CMV testing should be limited to infants with symmetric IUGR (i.e., weight and head circumference both affected). CMV testing at birth in very preterm (<32 weeks) and very low weight infants (<1500 g) may help to differentiate between congenital and postnatal CMV infection.

## Neonatal investigation, neonatal treatment and long-term follow-up

### Neonatal investigation and prognosis of neonatal infection

Following virological diagnosis of cCMV, investigations should assess organ involvement to predict outcome and guide treatment decision (Table 4).

#### Assessing the type and timing of maternal infection

MPI in the first trimester is a strong risk factor for sequelae.^7^ We recommend, when the type and the timing of maternal infection is unknown, to perform serology retrospectively on a stored serum from the first trimester, if available. For accurate timing of MPI, serology should be reviewed by a clinical virologist. MPI and MNPI have similar impact on the long-term outcomes of infants.^56^

#### Physical examination, laboratory investigation, ophthalmologic and audiologic assessment

Anthropometrics (weight, length and head circumference), physical examination, full blood count, liver enzymes, bilirubin (total and conjugated), ophthalmologic and audiologic assessment are required to classify the infection as symptomatic or asymptomatic (Table S8). The definition of “symptomatic” cCMV infection varies. Some studies include only physical examination findings, while others also consider abnormal laboratory results or neuroimagingfigure.^3^^,^^4^ Infants with cCMV and clinically-apparent disease at birth are more likely to have central nervous system (CNS) involvement, and 40–58% develop long-term neurological disabilities, including SNHL, intellectual disability, epilepsy, visual impairment and cerebral palsy.^2^ Infants with no apparent disease at birth have long-term sequelae in 13.5% of cases, mainly SNHL children.^2^

Microcephaly in relation to birth weight (i.e., HC *z* score—weight *z* score < −2) has a high specificity for poor neurological outcome (Table S10). Conversely, symmetric IUGR may not necessarily predict unfavorable outcome in infants without any other symptoms.

#### Neuroimaging assessment

While cranial ultrasound (cUS) is the first-line imaging modality, MRI may demonstrate significant pathology often missed by cUS (i.e., white matter abnormalities and cortical malformation). MRI is recommended in infants who present with clinical manifestations of CMV at birth, SNHL, chorioretinitis or abnormalities detected on cUS. MRI could also be undertaken in cases of MPI during the first trimester, or where timing of maternal infection is not known. There is no consensus on whether MRI should be performed in other situations.

Neuroimaging is the most reliable indicator of CNS involvement (Table S10). Normal neuroimaging predicts normal or near-normal neurodevelopmental outcome, while major lesions are associated with a poor prognosis (Table S6^57^, ^58^, ^59^, ^60^, ^61^). The prognostic implications of less severe neuroimaging findings (isolated lenticulostriate vasculopathy or subtle white matter abnormalities) are not yet fully understood.^61^ Combine cUS and MRI allows a comprehensive assessment with neuroimaging scoring systems (Table S6).

#### Virological factors

Overall, a neonatal high blood viral load (VL) correlates with symptomatic disease and sequelae, mainly SNHL, and a low or undetectable blood VL is associated with a lower risk of long term-sequelae (Table S11). However, there is no consensus on a blood VL threshold for risk stratification.

The rate of CMV DNA detection in CSF is low, even with confirmed CNS involvement (∼13–15%).^57^^,^^62^^,^^63^ Therefore, we do not recommend obtaining CSF specifically for diagnosis or assessment of cCMV.

### Neonatal infection treatment

Infants with significant symptoms/signs of cCMV can be treated with antivirals (intravenous ganciclovir, or more commonly its oral pro-drug valganciclovir). Two relatively small RCTs in symptomatic neonates^64^^,^^65^ showed modest benefits in terms of preservation of hearing and improved neurodevelopmental scores at 24 months of age. Comparison of 6 weeks versus 6 months of valganciclovir suggested greater efficacy with longer treatment.^65^ In both RCTS, infants were started on treatment before 1 month of age.

Whether starting later reduces efficacy of treatment remains uncertain. A non-randomized trial where infants with SNHL and no other clinical manifestation of cCMV started 6 weeks treatment up to 13 weeks of age did show improved preservation of hearing at 20 months of age.^66^ A RCT where children with SNHL aged 1 month to 4 years (median 13 months) were treated by 6 weeks by valganciclovir did not show any hearing improvement 6 months later.^67^

Cases of clinically significant isolated hepatitis, or isolated thrombocytopenia are rare. However, our expert opinion is to treat them for 6 weeks. We do not recommend treatment in isolated IUGR.

Chorioretinitis is normally associated with other symptoms and/or additional CNS involvement, and we recommend treatment for 6 months.

Due to poor recruitment, subsequent RCTs in asymptomatic children have not been completed and no RCTs included premature infants (<32 weeks’).

None of the RCTs considered brain imaging at randomization. Based on validated neuroimaging scoring (Table S6), infants with imaging scores 2 or 3 generally justify treatment. For score 1, expert advice should be sought.

Table S12 provides practical considerations for infants on treatment. Parents should be informed of the risks-benefit ratio of treatment. As valganciclovir/ganciclovir are not licensed for treatment of cCMV, all cases should be discussed with a Pediatric Infectious Diseases expert, and consented enrolment in the International CCMV Registry should be offered to parents (https://ccmvnet.org/). Cases may also be referred to the monthly CCMVNET Virtual Clinic for discussion (ccmvnet@gmail.com).

### Post-natal follow up

#### Ophthalmologic and neurodevelopmental follow-up

Recommendations for the follow-up of newborns diagnosed with cCMV have been previously published.^3^^,^^4^ The most noteworthy updates from the previous 2017 European guidelines are as follows.

- Ophthalmological follow-up is only recommended for those infants with retinitis at birth and not required for newborns with normal retinal examination.^68^
- Formal neurodevelopmental assessment at 24–36 months is recommended for children at risk for long-term sequelae (i.e., infection during first trimester or of unknown timing, apparent manifestations at birth, SNHL, chorioretinitis or presence of neuroimaging abnormalities). A recent systematic review found a cumulative incidence of neurodevelopmental impairment of 30–66% in symptomatic neonates. However asymptomatic infants performed equally well on neurodevelopmental assessments when compared with healthy controls. The authors highlighted that better long-term prospective studies, to school age, are required to clarify more subtle developmental outcomes.^69^ A recent study suggests that autism, attention-deficit/hyperactivity and behavioral problems may have a higher incidence among children with cCMV particularly those infected in the first trimester.^70^ This confirms the importance of monitoring until school entry to identify neurodevelopmental, behavioral, learning and late hearing problems in high-risk children. Children with cCMV without the above-mentioned risk factors for sequelae may follow standard pediatric care.

#### Hearing follow-up

In a prospective study of infected children screened at birth, 12.7% had SNHL at birth and 4.5% had delayed SNHL. Progressive SHNL is frequent (>50%) and children with unilateral SNHL at birth are at risk of developing SNHL in the contralateral ear.^71^

A meta-analysis based on prospective studies, reported 0.1% and 0% risk of SNHL in 226 children infected following a MPI in the second or third trimester respectively.^7^ A retrospective study showed a 2.1% (3/140) risk of late-onset SNHL in children born after a MPI in the second trimester.^72^ The risk of developing delayed SNHL after the age 5 years is not different in cCMV asymptomatic children compared to uninfected children.^73^ Therefore, based on current evidence, infected children should have regular hearing follow up to at least 5 years of age. Those with SNHL will require ongoing audiological follow-up. However, the estimated risk of delayed SNHL for asymptomatic children without SNHL at birth and with a proven MPI in the second trimester is low, and there is no full consensus on whether these cases need hearing follow-up. In children with proven MPI in the third trimester and normal hearing at birth, we do not recommend hearing follow-up.^74^

For the follow-up and rehabilitation of children with SNHL, we refer to relevant international guidelines.

#### Vestibular testing

The incidence and impact of vestibular damage is not completely understood. With a prevalence of 17% in cCMV children, recent publications suggest an incidence of vestibular problems as high (or higher) than SNHL.^75^^,^^76^ Vestibular problems can be congenital or delayed in onset, can improve, fluctuate or deteriorate and can be mild or severe.^75^ The impact of abnormal vestibular testing is not yet clear, but with early-onset and bilateral areflexia, the impact on early motor development is significant with greater risk for developmental delay.^75^ Identified vestibular risk factors are first trimester or unknown timing of maternal infection, SNHL and periventricular cysts on MRI.^75^

As yet, no consensus algorithm exists for follow-up of vestibular problems in children with cCMV. Based on the currently available evidence, we advise, when the facilities are available, vestibular screening at 6–8 months of age in high-risk children and those with abnormal motor development.^75^

#### Peer support

Families of children with cCMV should be informed about local and national peer support organizations, as well as websites with reliable and accessible information. A new diagnosis of cCMV in the prenatal or neonatal period, with all the uncertainties for the future, can be very difficult for parents to process. Both peer and clinical psychological support may be very helpful for families on the early steps of the journey.

## Conclusion

This article reflects the collective viewpoint of a cohort of experts who share a specific interest in cCMV. Compared to previous published guidelines, the main innovations are the following.

Maternal CMV serology should be performed in the first trimester of pregnancy, as cCMV sequelae are limited to maternal infection acquired in the first trimester of pregnancy.

In cases of MPI in the periconceptional period or in the first trimester, oral valaciclovir at a dose of 8 g/day should be administered as early as possible after the diagnosis and until the amniocentesis.

A negative CMV PCR in amniotic fluid following timely amniocentesis ensures absence of long-term sequelae.

Newborns with CNS-related symptoms but also those with isolated SNHL, should be treated with valganciclovir. Treatment should be started as soon as possible and before 1 month of age. Treatment initiated between 1 and 3 months may be beneficial in those children with SNHL.

Children with cCMV and confirmed transmission in the first trimester or unknown timing of transmission should be followed up to at least 6 years of age to ensure specialized management. For those with documented MPI in the second and third trimesters of pregnancy this follow-up may not be necessary.

## Contributors

All authors independently searched the literature and assessed the eligibility of all identified citations. All authors participated in the extraction of the data from included studies. MLV and CC performed the assessment of bias and GRADE evaluation. All authors contributed to the interpretation of the results, provided critical feedback and helped shape the manuscript.

## Declaration of interests

MLV reports receiving support for attending meetings by BioMérieux and Altona outside the submitted work. MLV reports payment made to her institution for presentation/lecture by Diasorin, Abbott Molecular and Roche Diagnostic outside the submitted work. DBG received Grant from the Spanish Ministry of Science and Innovation. ISCIII and Fondos FEDER (EU) outside the submitted work and received Honoraria for lectures from the Medscape, outside the submitted work. JF received honoraria for poster presentation by Abbott GmbH, outside the submitted work. CJ received consulting fees from Moderna, outside the submitted work; Participated on a Data Safety Monitoring Board or Advisory Board of Moderna, outside the submitted work; Is Co-director of the European Congenital CMV Initiative; Her institution received payment for clinical trials by Moderna, outside the submitted work. HL participated on advisory board for the Study of Zidovudine in children with Aicadri-Goutiere Syndrome, outside the submitted work and she is Trustee of European Society For Paediatric Infectious Diseases (ESPID). VP institution received honoraria for lectures from MSD and Pfizer, outside the submitted work and received support for attending a meeting (ESPID 2023) from Pfizer. PV is president and board-member of the European Congenital CMV Initiative. AV is participating in the Advisory Council European Society of Virology. The rest author of the authors declare no conflict of interest.
