# Corrigendum to “Consensus recommendation for prenatal, neonatal and postnatal management of congenital cytomegalovirus infection from the European congenital infection initiative (ECCI)” [The Lancet Regional Health – Europe 40 (2024) 100892]

**DOI:** 10.1016/j.lanepe.2024.100974

**Published:** 2024-06-24

**Authors:** Marianne Leruez-Ville, Christos Chatzakis, Daniele Lilleri, Daniel Blazquez-Gamero, Ana Alarcon, Nicolas Bourgon, Ina Foulon, Jacques Fourgeaud, Anna Gonce, Christine E. Jones, Paul Klapper, André Krom, Tiziana Lazzarotto, Hermione Lyall, Paulo Paixao, Vassiliki Papaevangelou, Elisabeth Puchhammer, George Sourvinos, Pamela Vallely, Yves Ville, Ann Vossen

**Affiliations:** a Université Paris Cité, URP 7328 FETUS, Paris F-75015, France; b Virology Laboratory, Reference Laboratory for Cytomegalovirus Infections, Hôpital Necker Enfants Malades, GHU Paris Centre, AP-HP, Paris, France; c Obstetrics, Fetal Medicine Surgery and Imaging Unit, Hôpital Necker Enfants Malades, GHU Paris Centre, AP-HP, Paris, France; d Second Department of Obstetrics and Gynecology of Aristotle University of Thessaloniki, Thessaloniki, Greece; e Microbiology and Virology Unit, Fondazione IRCCS Policlinico San Matteo, Pavia, Italy; f Pediatric Infectious Diseases Unit, Hospital Universitario 12 de Octubre, Instituto de Investigación Hospital 12 de Octubre (imas12), Universidad Complutense, Madrid, Spain; g Department of Neonatology, Hospital Hospital Sant Joan de Déu, BCNatal (Barcelona Center for Maternal, Fetal and Neonatal Medicine), Institut de Recerca Sant Joan de Déu, Universitat de Barcelona, Barcelona, Spain; h Department of Otorhinolaryngology and Head & Neck Surgery, Vrije Universiteit Brussels, University Hospital UZ Brussel, Brussels Health Campus, De Poolster, Rehabilitation Centre, Brussels, Belgium; i BCNatal: Fetal Medicine Research Center (Hospital Clínic and Hospital Sant Joan de Déu), Universitat de Barcelona, Barcelona, Spain; j Faculty of Medicine and Institute for Life Sciences, University of Southampton and NIHR Southampton Clinical Research Facility and NIHR Southampton Biomedical Research Centre, University Hospital Southampton NHS Foundation Trust, United Kingdom; k Microbiology and Virology Unit (EIGen), School of Biological Sciences, University of Manchester, Manchester, M139PT, UK; l Department of Medical Ethics and Health Law, Leiden University Medical Center, Leiden, the Netherlands; m Microbiology Unit, IRCCS Azienda Ospedaliero-Universitaria di Bologna, Bologna, Italy; n Department of Medical and Surgical Sciences (DIMEC), University of Bologna, Bologna, Italy; o Imperial College Healthcare NHS Trust, London, England; p CHRC, NOVA Medical School/Faculdade de Ciências Médicas, Universidade NOVA de Lisboa, Lisbon 1169-056, Portugal; q Third Department of Pediatrics, School of Medicine, National and Kapodistrian University of Athens, Greece; r Center for Virology, Medical University Vienna, Austria; s Laboratory of Clinical Virology, Medical School, University of Crete, Heraklion, Crete 71003, Greece; t Department of Medical Microbiology, Leiden University Medical Center, Leiden, the Netherlands

The authors have noticed an error in the title where it should read “European congenital cytomegalovirus initiative (ECCI)” instead of “European congenital infection initiative (ECCI)”.

The authors would like to apologise for any inconvenience caused.

