# Supplementary material for: Consensus recommendation for prenatal, neonatal and postnatal management of congenital cytomegalovirus infection from the European congenital infection initiative (ECCI)
Source: Lancet Reg Health Eur. 2024 Apr 1;40:100892. doi: 10.1016/j.lanepe.2024.100892 (PMC10999471; doi:10.1016/j.lanepe.2024.100892)
Supplement: Supplementary data [file mmc1.docx]

**Supplementary data**

1. **Recommendations workshop document**

The key questions that were addressed by the attendees of the cCMV recommendations workshop were the following.

1. **Primary prevention and awareness**
2. Should primary prevention by hygienic measures be recommended to avoid maternal primary infection?
3. Should primary prevention by hygienic measures be recommended to avoid maternal non-primary infection?
4. What is the awareness of cCMV in the general population, in pregnant women and in health care?
5. Who should be targeted for educational programs and how?
6. **Diagnosis of maternal CMV infection**
7. How do CMV IgG last generation commercial assays compare?
8. When should you prescribe a CMV serology in pregnancy?
9. What is the sensitivity of CMV IgM last generation commercial assays for the diagnosis of CMV primary infection?
10. What is the specificity of CMV IgM last generation commercial assays for the diagnosis of CMV primary infection?
11. What is the sensitivity of CMV IgG avidity last generation commercial assays to exclude a recent primary infection?
12. Is CMV DNA detection/quantification in maternal blood and urine useful for the diagnosis of primary infection in pregnant women?
13. Are CMV-IgG kinetics or CMV-IgM detection useful for diagnosis of maternal non-primary infection?
14. Is CMV-DNA detection/quantification in maternal blood and urine useful for the diagnosis of non-primary infection in pregnant women?
15. **Secondary prevention, diagnosis of fetal infection and follow-up of infected fetuses**
16. Should valaciclovir prevention be used in pregnant women with a primary-infection? If so, what clinical settings are appropriate? What clinical settings are not appropriate? What is the best regimen?
17. Should hyperimmune globulin be used in pregnant women with a primary infection?

If so, what clinical settings are appropriate? What clinical settings are not appropriate? What is the best regimen?

1. When is the best timing for diagnosis of fetal infection by CMV PCR in amniotic fluid in case of maternal primary-infection?
2. What is the proportion and the prognosis of infants with cCMV and a negative CMV PCR in amniotic fluid at amniocentesis?
3. What are the options for antiviral treatment of infected fetuses?
4. **Neonatal diagnosis**
5. What is the best timing for neonatal diagnosis?
6. What samples should be used for neonatal diagnosis ?

Urine? Blood? Saliva?

1. What is the performance of CMV PCR in dried-blood spots for cCMV retrospective diagnosis?
2. Which neonates should benefit from a cCMV neonatal diagnosis?
3. **Neonatal investigation, neonatal treatment and long-term follow-up**
4. What is the definition of a symptomatic and an asymptomatic infant with cCMV?
5. What is the prognosis value of neonatal clinical features and neonatal imaging?
6. What is the utility of surrogate virological biomarkers for prognosis?
7. Should off-label antiviral agents be used for neonates? If so, what is the rationale? What clinical settings are appropriate? What clinical settings are not appropriate? What is the best regimen? (Oral vs IV vs combined, duration, etc.)
8. What standard definitions should be used in clinical decision making for antiviral therapy?
9. Which infected neonates should benefit from long term-follow-up?
10. What investigations should long-term follow comprise and how long should this long-term follow-up be?
11. **Risk of bias assessment of the included studies**
12. **Primary prevention and awareness**

**Recommendation A.1**

| ***Assessment of risk of bias of study assessing the efficacy of primary prevention by hygienic measures using risk of bias tool 2*** | | | | | | | |
| --- | --- | --- | --- | --- | --- | --- | --- |
|  | 1 | 2 | 3 | 4 | 5 | 6 | Overall |
| Adler 2004 ^1^ | 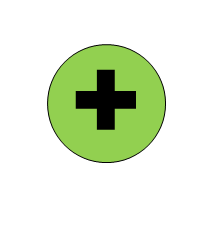 | 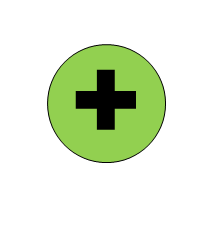 | 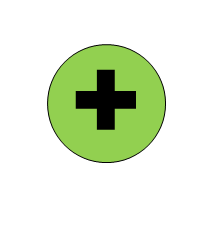 | 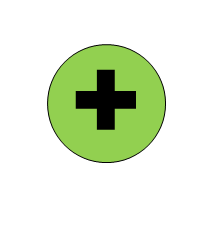 | 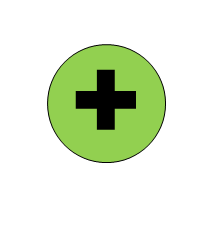 | 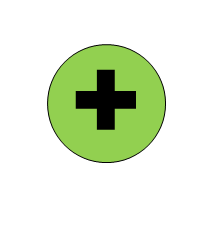 | Low |

1. Bias arising from the randomization process
2. Bias due to deviations from intended interventions (effect of assignment to intervention)
3. Bias due to deviations from intended interventions (effect starting and adhering to intervention)
4. Bias due to missing outcome data
5. Bias in measurement of the outcome
6. Bias in selection of the reported result

**Assessment of risk of bias of study assessing the efficacy of primary prevention by hygienic measures using Newcastle Ottawa Scale**

| **Studies** | **Selection** | **Comparability** | **Outcome** | **Total stars** |
| --- | --- | --- | --- | --- |
| Vauloup-Fellous 2009 ^2^ | **** | ** | ** | 8 |
| Revello 2015 ^3^ | **** | ** | ** | 8 |

**Recommendation A2**

**Assessment of risk of studies assessing the awareness of cCMV in women of child bearing age and the risk of primary infection according to the interval between pregnancy bias using Newcastle Ottawa Scale**

| **Studies** | **Selection** | **Comparability** | **Outcome** | **Total stars** |
| --- | --- | --- | --- | --- |
| Leruez-Ville 2020 ^4^ | **** | ** | ** | 8 |
| Binda S, 2016 ^5^ | **** |  | ** | 6 |
| Fowler K et al, 2004 ^6^ | *** | ** | ** | 7 |

**Recommendation A3a**

**Assessment of risk of bias assessing the awareness of cCMV among healthcare professionals using Newcastle Ottawa Scale**

| **Studies** | **Selection** | **Comparability** | **Outcome** | **Total stars** |
| --- | --- | --- | --- | --- |
| Fellah 2020 ^7^ | **** | ** | ** | 8 |
| Castillo 2022 ^8^ | **** | ** | ** | 8 |

1. **Diagnosis of maternal CMV infection**

**Recommendation B.3a**

**Assessment of the risk of bias of studies reporting sensitivity and specificity of IgM testing for the diagnosis of primary infection in pregnancy using QUADAS-2 (Quality Assessment of Diagnostic Accuracy Studies).**

| Study | Risk of bias | | | |
| --- | --- | --- | --- | --- |
|  | Patient selection | Index test | Reference standard | Flow and timing |
| Carlier 2010 ^9^ | 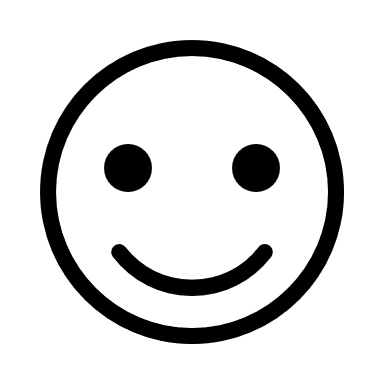 | 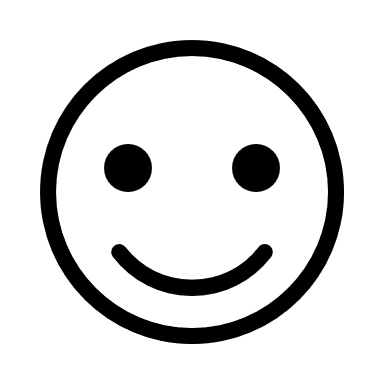 | 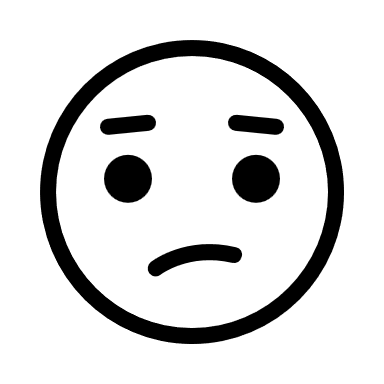 | 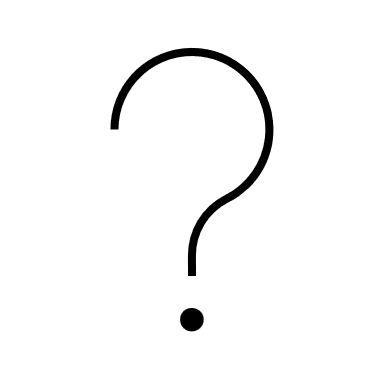 |
| Chiereghin 2017 ^10^ | 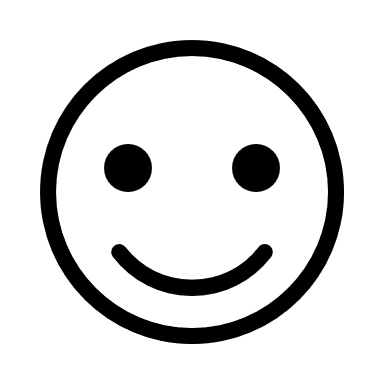 | 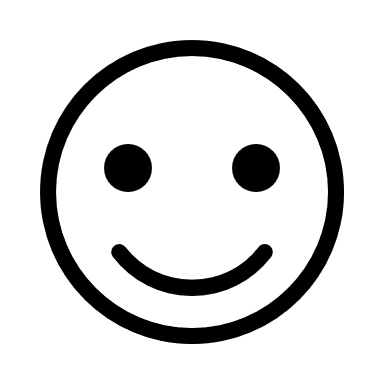 | 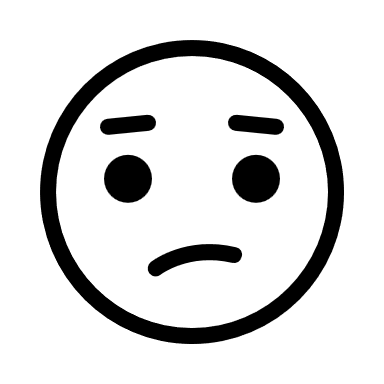 | 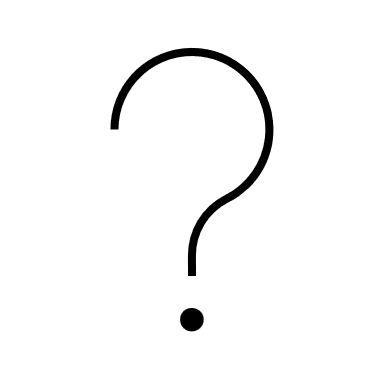 |
| Delforge 2015 ^11^ | 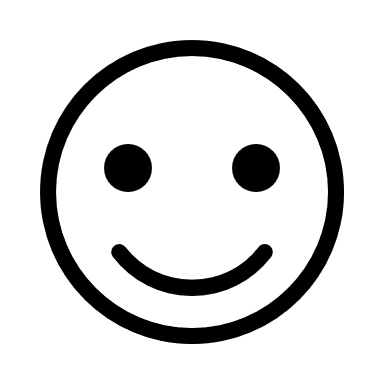 | 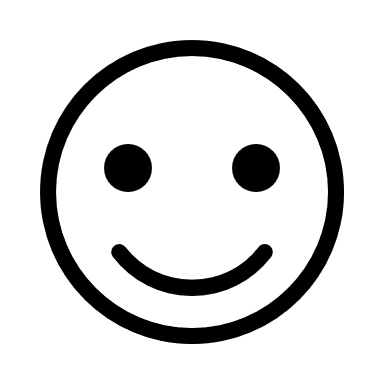 | 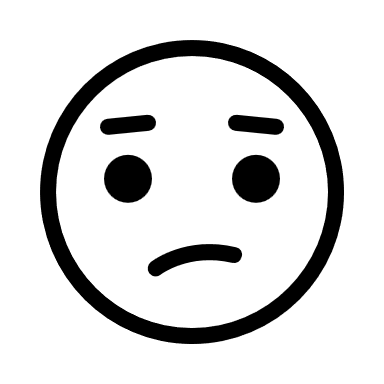 | 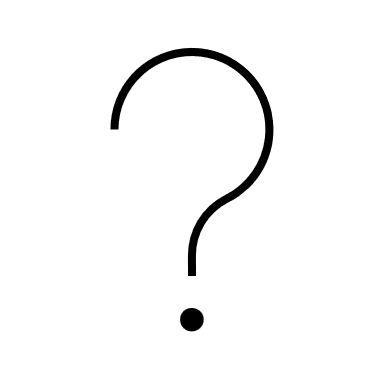 |
| Genco 2019 ^12^ | 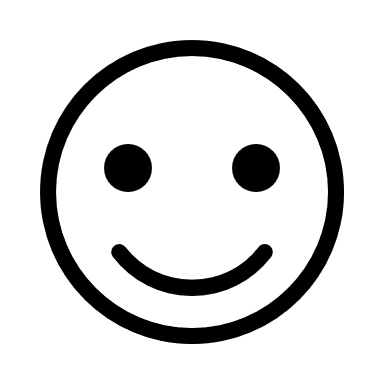 | 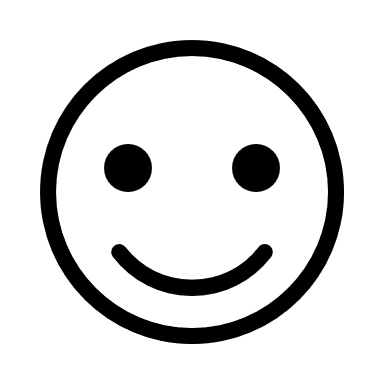 | 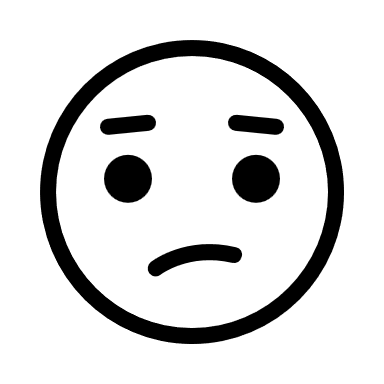 | 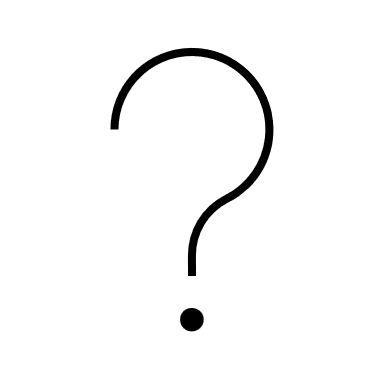 |
| Revello 2012 ^13^ | 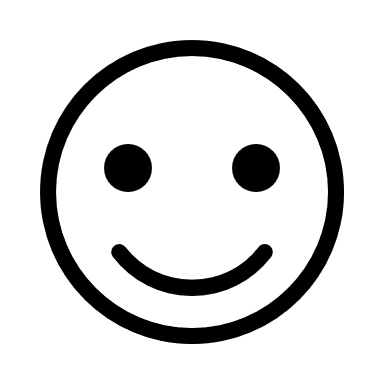 | 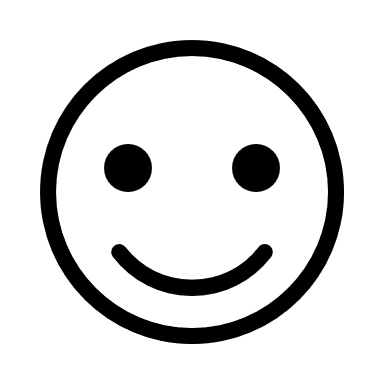 | 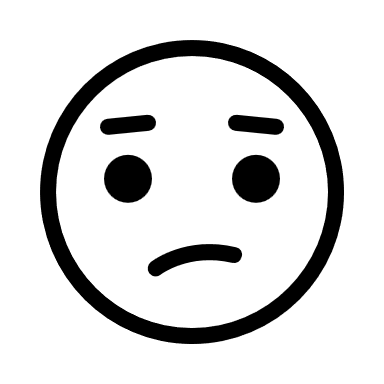 | 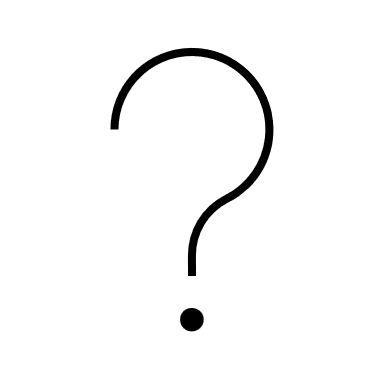 |
| Sarasini 2021 ^14^ | 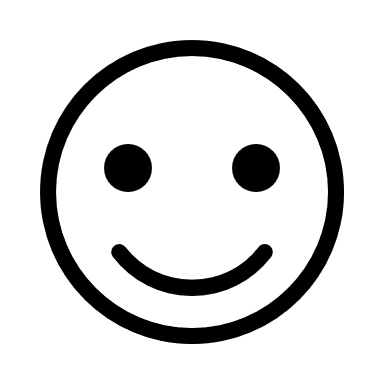 | 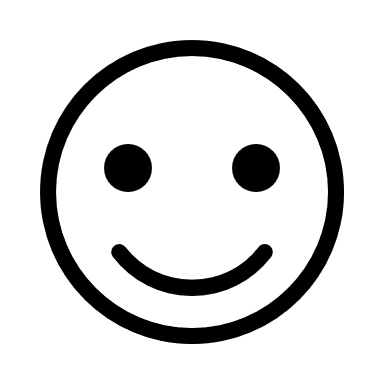 | 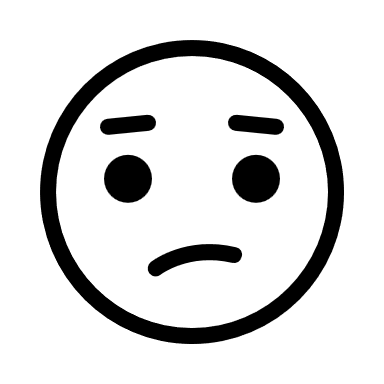 | 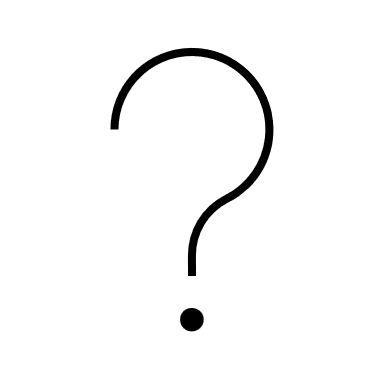 |


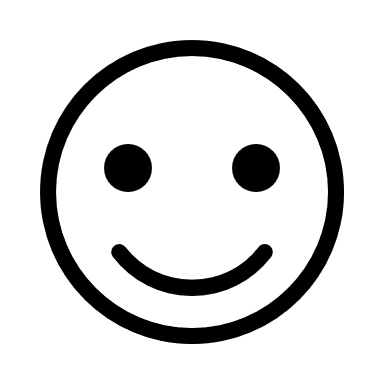
Low Risk
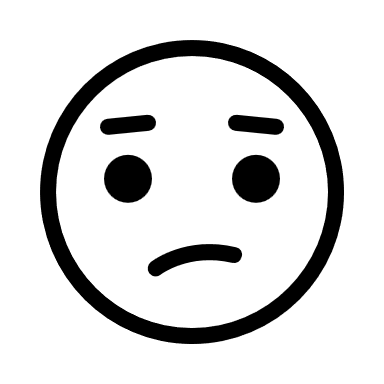
High Risk
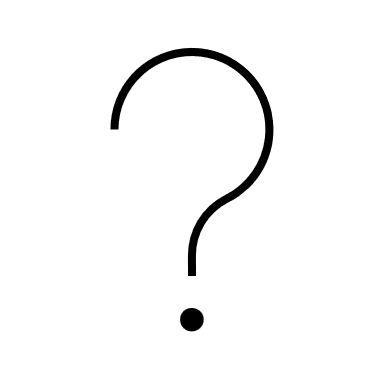
Unclear Risk

QUADAS-2 (Quality Assessment of Diagnostic Accuracy Studies) is a tool widely used for assessing the risk of bias in diagnostic accuracy studies. It evaluates four key domains: patient selection, index test, reference standard, and flow and timing. Each domain is assessed for concerns regarding bias, applicability, and signaling questions. The assessment involves evaluating factors such as the appropriateness of participant inclusion criteria, blinding of index and reference tests, and the potential for verification bias. Additionally, the tool considers the adequacy of the interval between tests and the completeness of data.

All the included studies were characterized as high risk for bias in the reference standard domain due to the absence of reference standard. In addition, all the studies were characterized as unclear risk of bias in the flow and timing domain, as the interval between index and reference standard is not applicable, due to the absence of reference standard.

**Recommendation B.3b**

**Assessment of risk of bias of studies reporting the performance of avidity assays to exclude a recent primary infection using QUADAS-2 (Quality Assessment of Diagnostic Accuracy Studies).**

| Study | Risk of bias | | | |
| --- | --- | --- | --- | --- |
|  | Patient selection | Index test | Reference standard | Flow and timing |
| Chiereghin 2017 ^10^ | 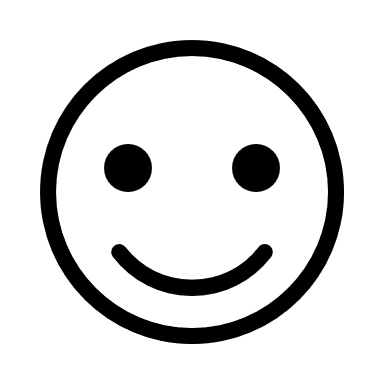 | 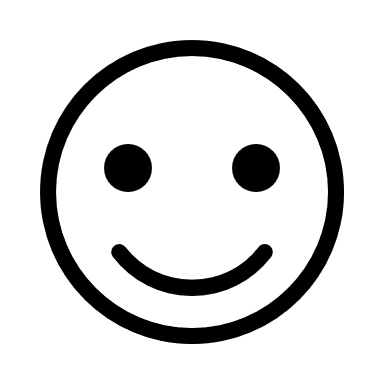 | 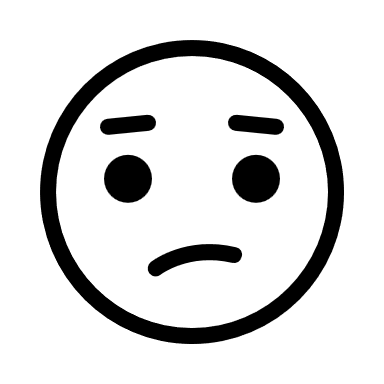 | 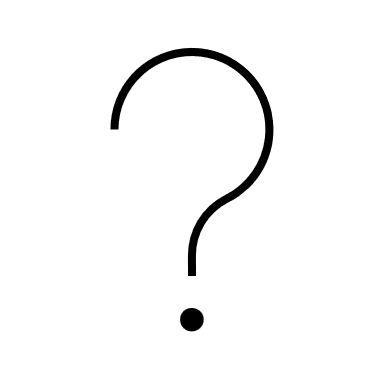 |
| Delforge 2015 ^11^ | 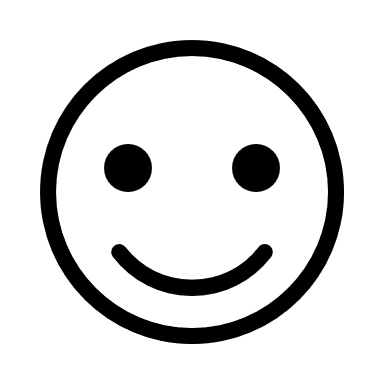 | 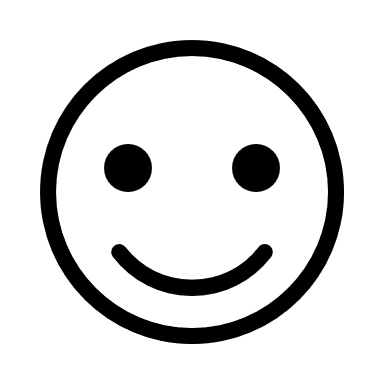 | 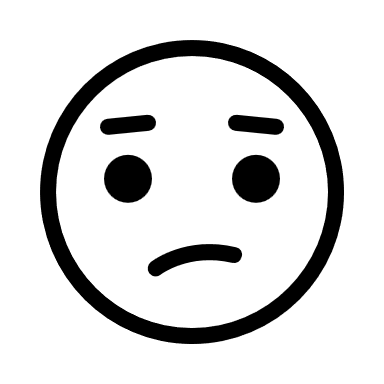 | 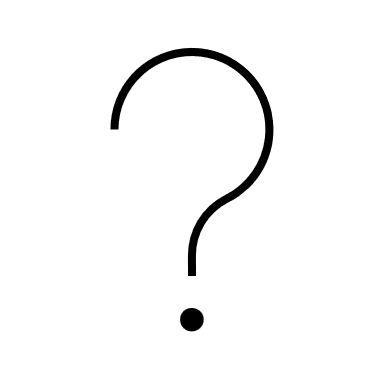 |
| Lagrou 2009 ^15^ | 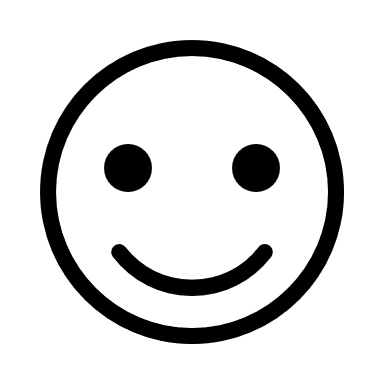 | 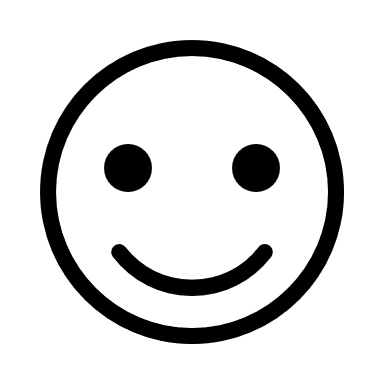 | 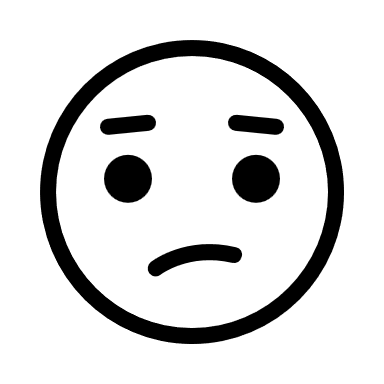 | 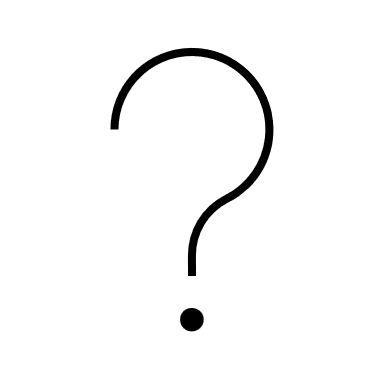 |
| Sarasini 2021 ^15^ | 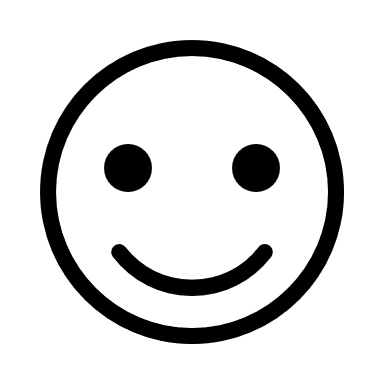 | 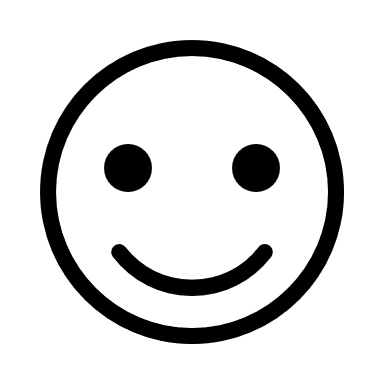 | 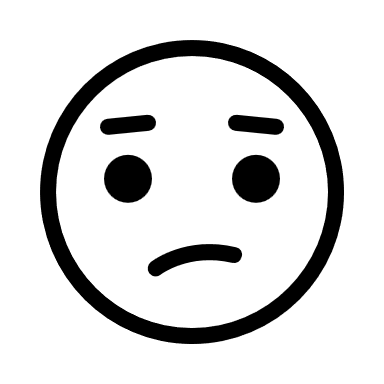 | 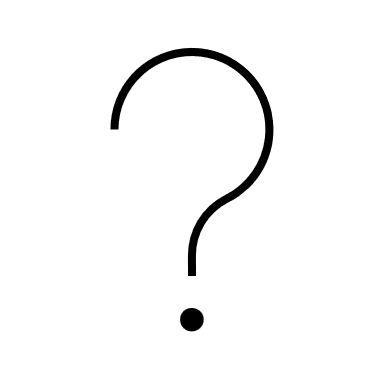 |
| Vauloup-Fellous 2013 ^16^ | 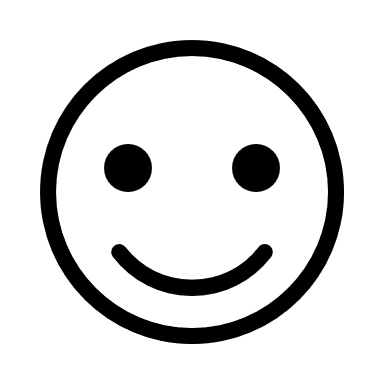 | 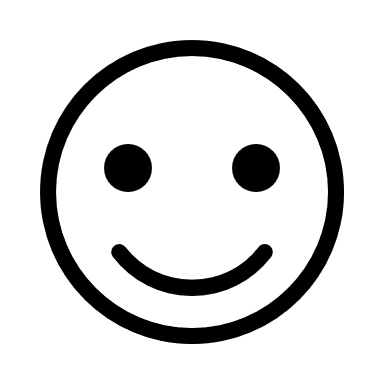 | 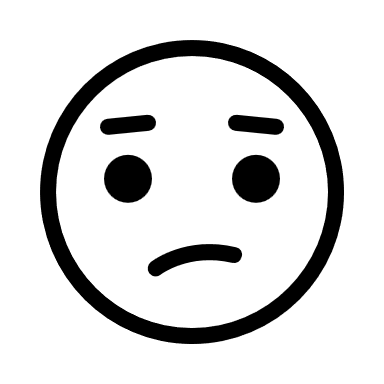 | 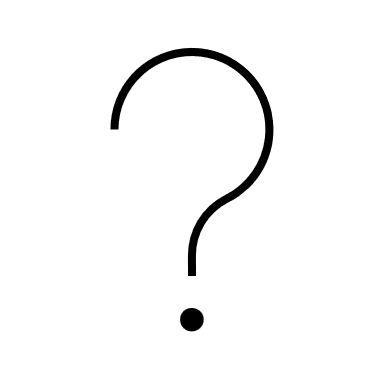 |
| Vauloup-Fellous 2014 ^17^ | 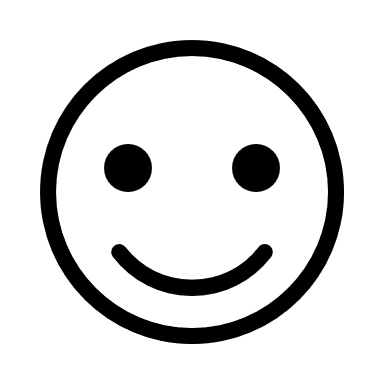 | 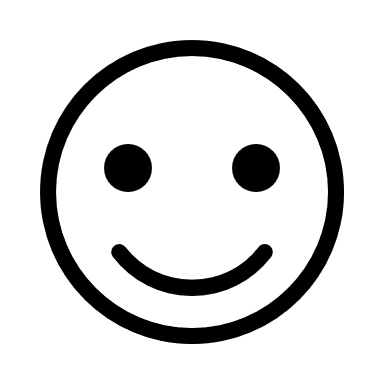 | 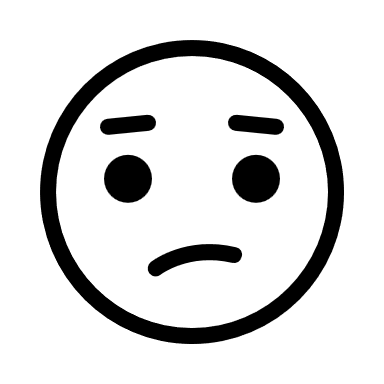 | 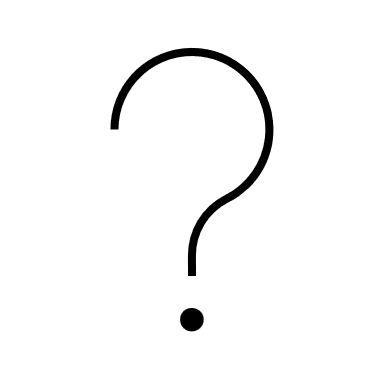 |


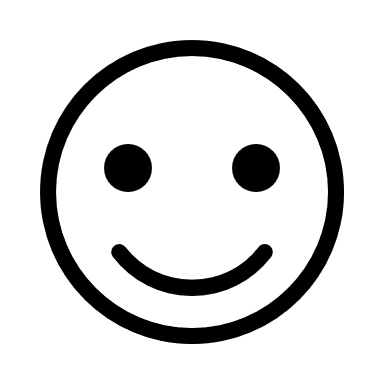
Low Risk
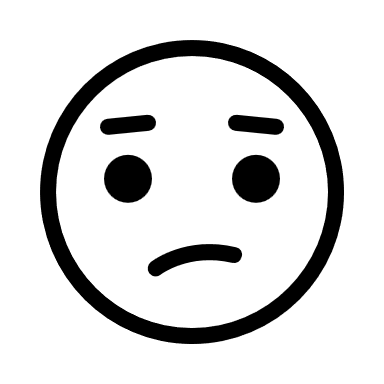
High Risk
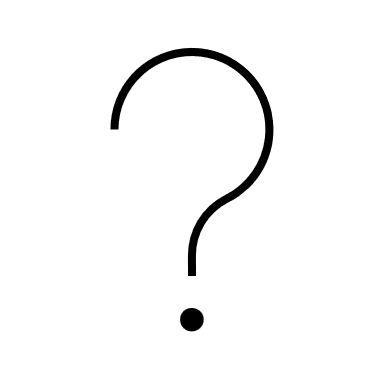
Unclear Risk

**Recommendation B.3c**

**Assessment of risk of bias of studies reporting the performance of CMV PCR in blood and urine for the diagnosis of recent primary infection using QUADAS-2 (Quality Assessment of Diagnostic Accuracy Studies).**

| Study | Risk of bias | | | |
| --- | --- | --- | --- | --- |
|  | Patient selection | Index test | Reference standard | Flow and timing |
| Berth 2016 ^18^ | 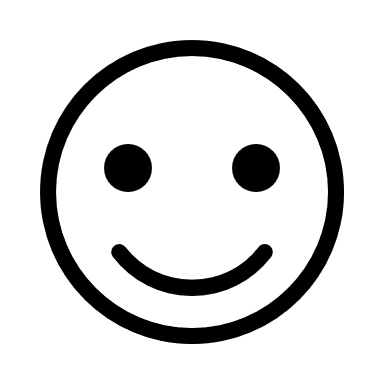 | 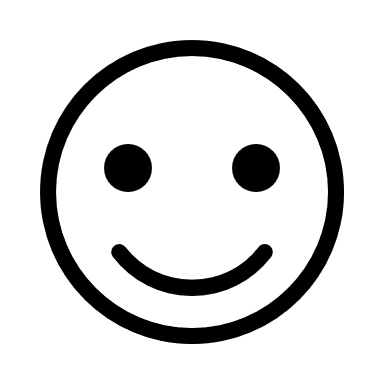 | 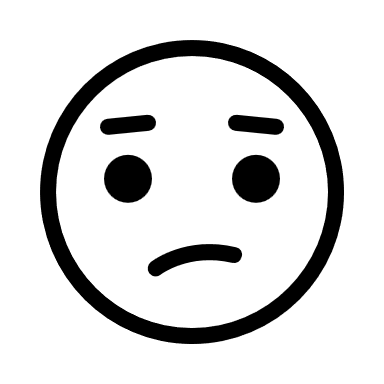 | 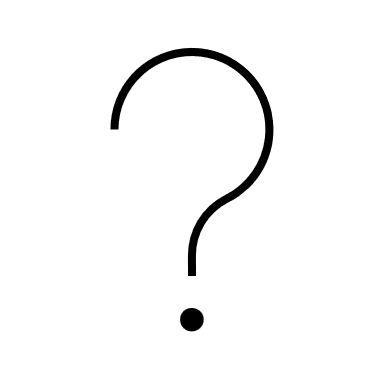 |
| Fornara 2022 ^19^ | 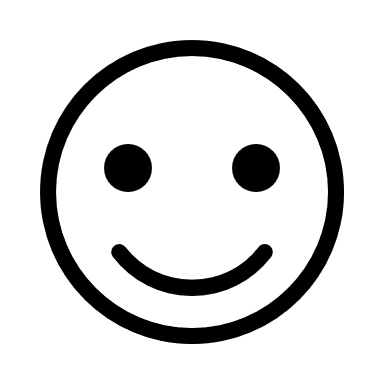 | 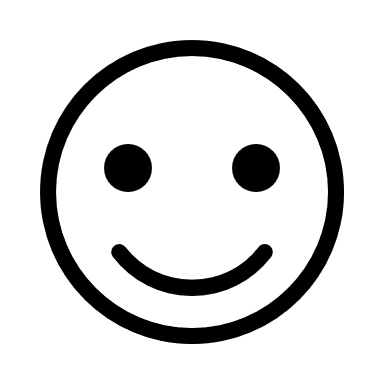 | 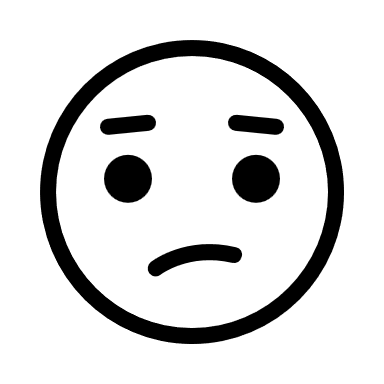 | 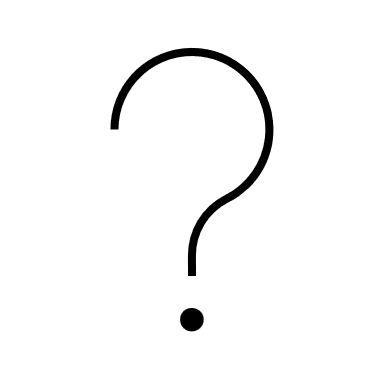 |
| Périllaud-Dubois 2022 ^20^ | 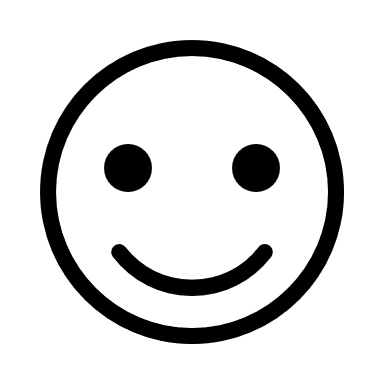 | 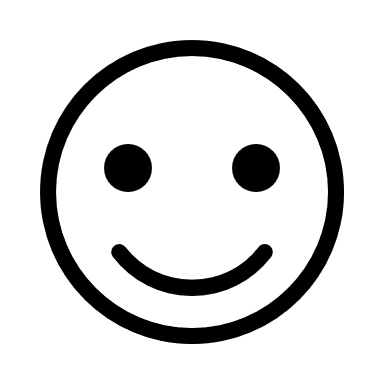 | 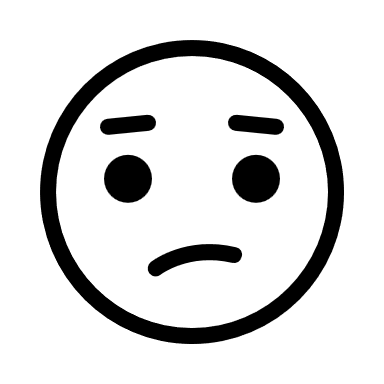 | 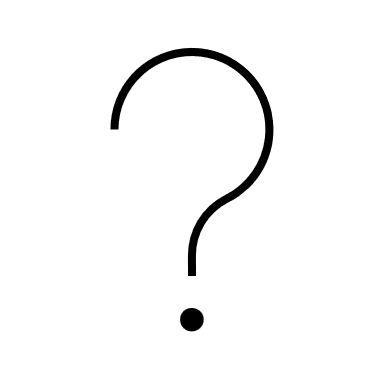 |
| Revello 2001 ^21^ | 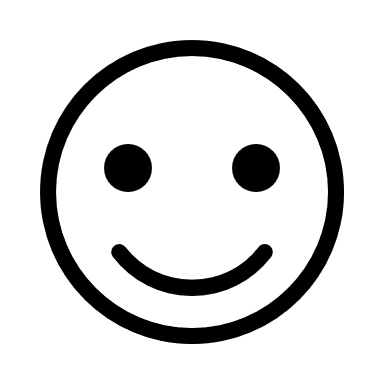 | 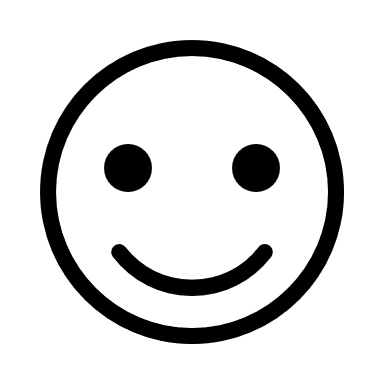 | 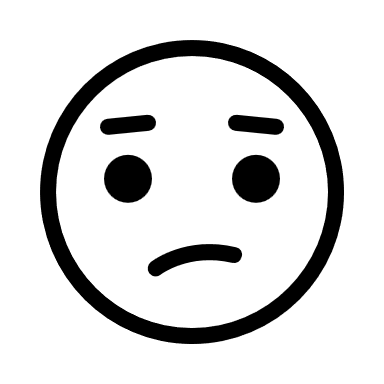 | 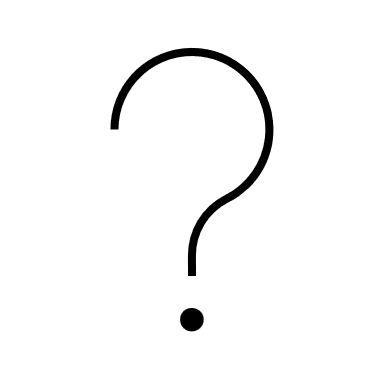 |
| Sarasini 2021 ^14^ | 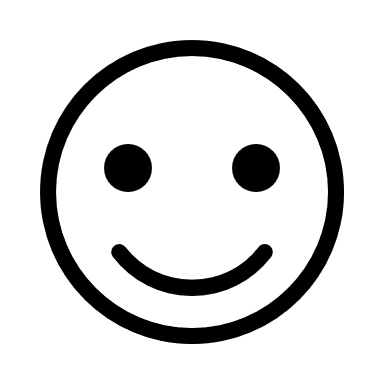 | 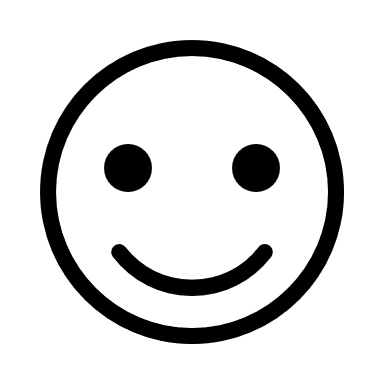 | 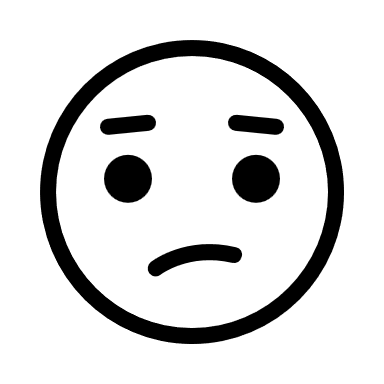 | 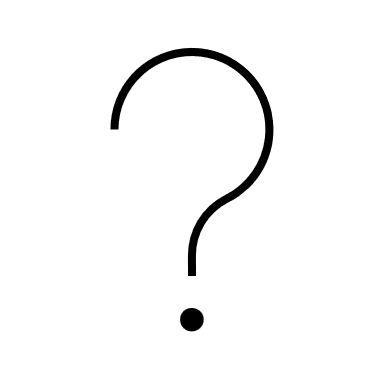 |


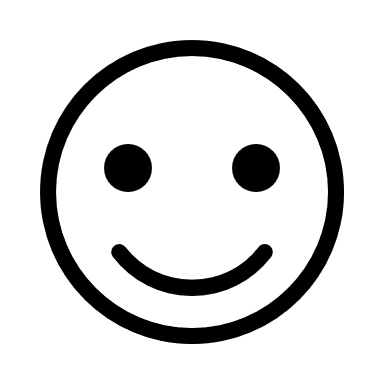
Low Risk
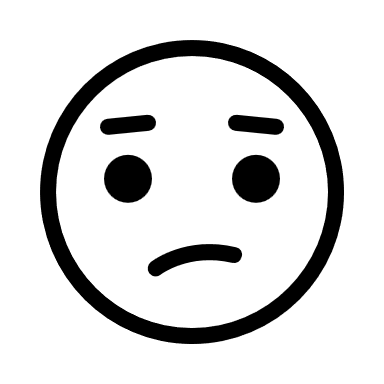
High Risk
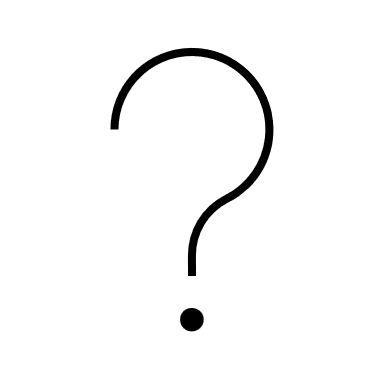
Unclear Risk

**Recommendation B.4**

**Assessment of risk of bias of studies reporting the performance of serology to identify among women with preexisting immunity those at risk of given birth to an infected neonate using QUADAS-2 (Quality Assessment of Diagnostic Accuracy Studies).**

| Study | Risk of bias | | | |
| --- | --- | --- | --- | --- |
|  | Patient selection | Index test | Reference standard | Flow and timing |
| Hadar 2017 ^22^ | 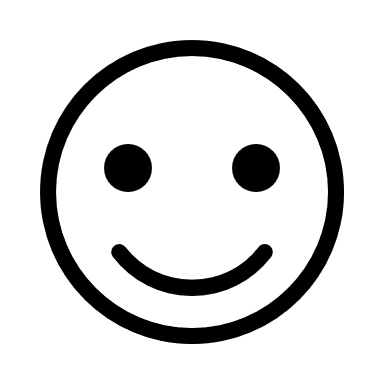 | 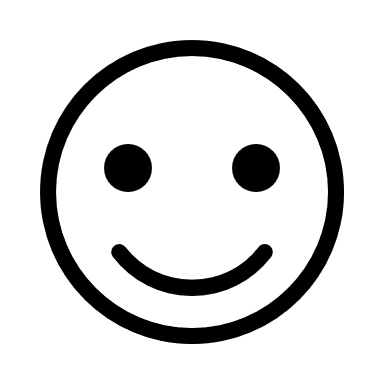 | 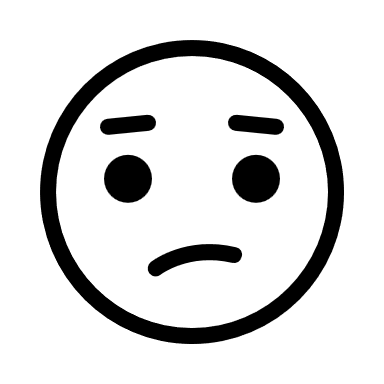 | 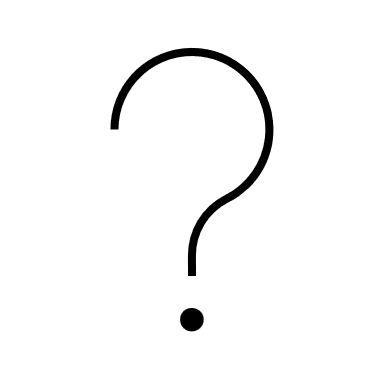 |
| Leruez-Ville 2017 ^23^ | 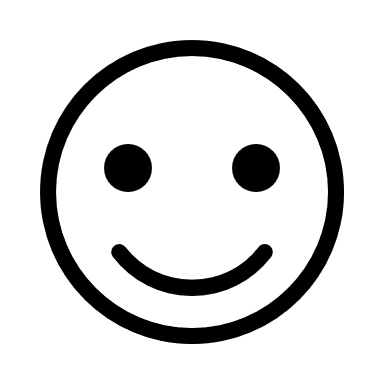 | 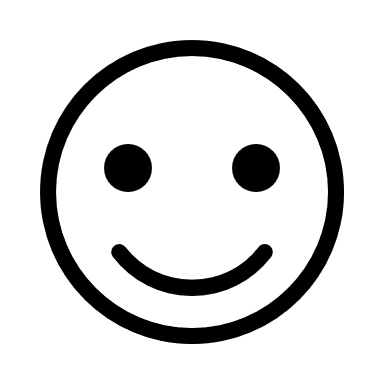 | 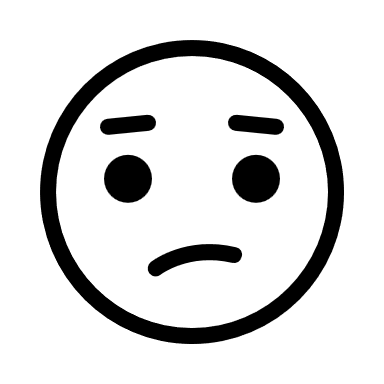 | 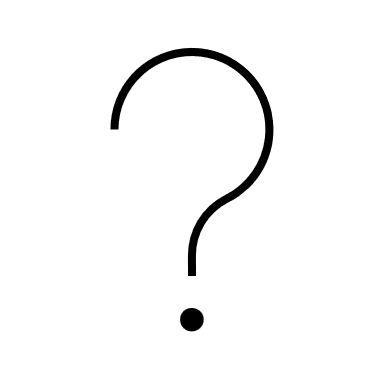 |
| Lilleri 2023 ^24^ | 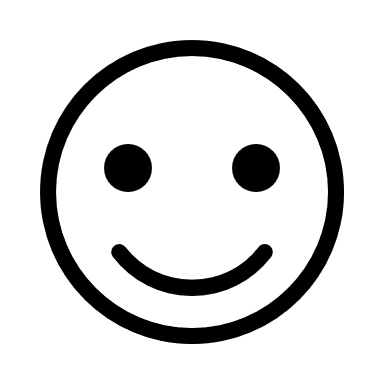 | 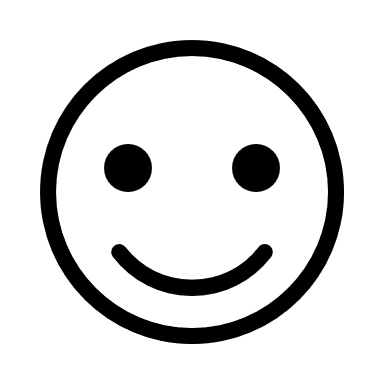 | 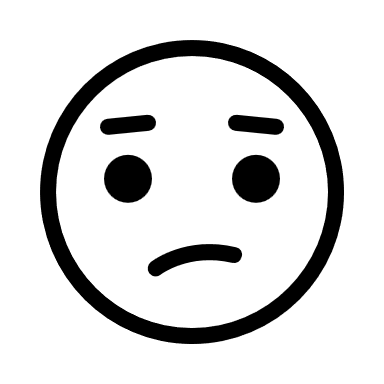 | 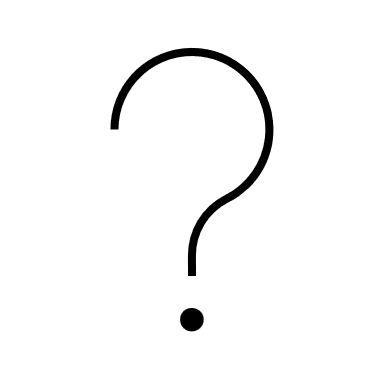 |
| Périllaud-Dubois 2022 ^25^ | 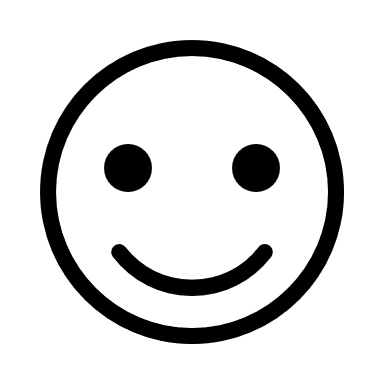 | 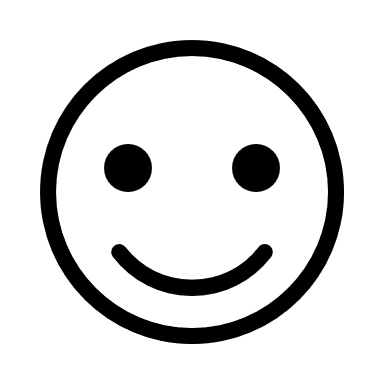 | 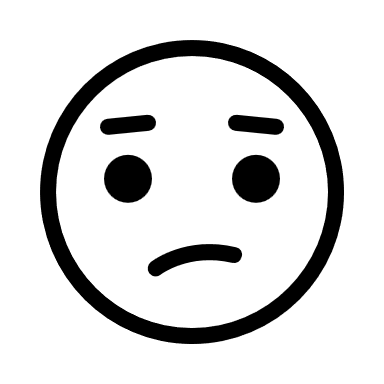 | 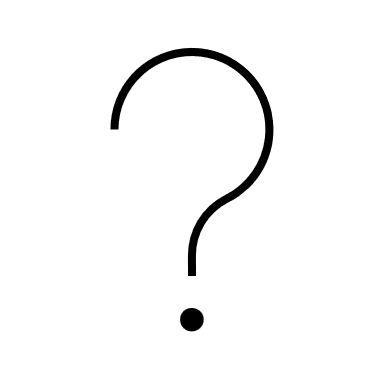 |
| Picone 2017 ^26^ | 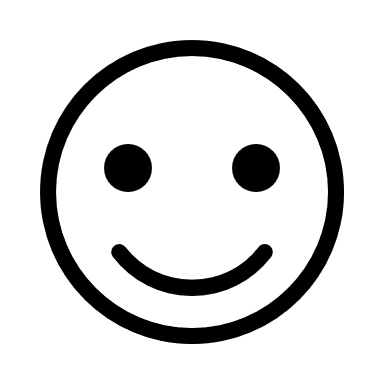 |  |  |  |
| Puhakka 2017 ^27^ |  |  |  |  |

Low Risk High Risk Unclear Risk

1. **Secondary prevention, diagnosis of fetal infection and follow-up of infected fetuses**

**Recommendation C.1a**

***Risk of bias of the Randomized controlled trial of secondary prevention by valacyclovir 8g/day using ROB 2 tool***

| **Study-author** | 1 |  | 2 | 3 | 4 | 5 | 6 | Overall |
| --- | --- | --- | --- | --- | --- | --- | --- | --- |
| Shahar-Nissan 2020 ^28^ |  |  |  |  |  |  |  | Low |

1. Bias arising from the randomization process.
2. Bias due to deviations from intended interventions (effect of assignment to intervention).
3. Bias due to deviations from intended interventions (effect starting and adhering to intervention).
4. Bias due to missing outcome data.
5. Bias in measurement of the outcome.
6. Bias in selection of the reported result.

**Risk of bias of non-randomized interventional studies of secondary prevention by valacyclovir 8g/day using Robins-I tool.**

| **Study-author** | **1** | **2** | **3** | **4** | **5** | **6** | **7** | **Overall risk of bias** |
| --- | --- | --- | --- | --- | --- | --- | --- | --- |
| Faure-Bardon 2022 ^29^ |  |  |  |  |  |  |  | Low risk |
| Egloff 2023 ^30^ |  |  |  |  |  |  |  | Low risk |

1. Bias due to confounding
2. Bias in selection of participants into the study
3. Bias in classification of interventions
4. Bias due to deviations from intended intervention
5. Bias due to missing data
6. Bias in measurement of outcomes
7. Bias in selection of the reported result

**Recommendation C.1a**

**Risk of bias of non-randomized interventional studies for the risk of acute renal failure depending on the dose regimen using Robins-I tool.**

| **Study-author** | **1** | **2** | **3** | **4** | **5** | **6** | **7** | **Overall risk of bias** |
| --- | --- | --- | --- | --- | --- | --- | --- | --- |
| Faure-Bardon 2021 ^29^ |  |  |  |  |  |  |  | Low risk |
| Egloff 2023 ^30^ |  |  |  |  |  |  |  | Low risk |
| Ville Y 2021 ^31^ |  |  |  |  |  |  |  | Some concerns |

1. Bias due to confounding
2. Bias in selection of participants into the study
3. Bias in classification of interventions
4. Bias due to deviations from intended intervention
5. Bias due to missing data
6. Bias in measurement of outcomes
7. Bias in selection of the reported result

**Recommendation C.1b**

***Risk of bias of Randomized controlled trial for secondary prevention with hyper-immune globulin 100IU/kg every 4 weeks using ROB 2 tool.***

|  | 1 | 2 | 3 | 4 | 5 | 6 | Overall |
| --- | --- | --- | --- | --- | --- | --- | --- |
| Revello 2014 ^32^ |  |  |  |  |  |  | Low |
| Hughes 2021 ^33^ |  |  |  |  |  |  | Low |

1. Bias arising from the randomization process.
2. Bias due to deviations from intended interventions (effect of assignment to intervention).
3. Bias due to deviations from intended interventions (effect starting and adhering to intervention).
4. Bias due to missing outcome data.
5. Bias in measurement of the outcome.
6. Bias in selection of the reported result.

**Risk of bias of non-randomized interventional study for secondary prevention with hyperimmune globulin 200IU/kg every 2 weeks, using Robins-I tool.**

| **Study-author** | **1** | **2** | **3** | **4** | **5** | **6** | **7** | **Overall risk of bias** |
| --- | --- | --- | --- | --- | --- | --- | --- | --- |
| Kagan 2019 ^34^ |  |  |  |  |  |  |  | Low risk |

1. Bias due to confounding
2. Bias in selection of participants into the study
3. Bias in classification of interventions
4. Bias due to deviations from intended intervention
5. Bias due to missing data
6. Bias in measurement of outcomes
7. Bias in selection of the reported result

**Recommendation C.2a**

***Assessment of risk of bias of studies evaluating the performance and the timing of CMV PCR in amniotic fluid for the diagnosis of fetal infection using QUADAS-2 (Quality Assessment of Diagnostic Accuracy Studies).***

| Study | Risk of bias | | | |
| --- | --- | --- | --- | --- |
|  | Patient selection | Index test | Reference standard | Flow and timing |
| Donner 1994 ^35^ |  |  |  |  |
| Revello 1995 ^36^ |  |  |  |  |
| Liesnard 2000 ^37^ |  |  |  |  |
| Enders 2001 ^38^ |  |  |  |  |
| Enders 2017 ^39^ |  |  |  |  |

Low Risk High Risk Unclear Risk

**Recommendation C.2b**

**Risk of bias of non-randomized interventional studies for the treatment with valacyclovir 8g per day for infected fetuses using Robins-I tool.**

| **Study-author** | **1** | **2** | **3** | **4** | **5** | **6** | **7** | **Overall risk of bias** |
| --- | --- | --- | --- | --- | --- | --- | --- | --- |
| Leruez-Ville 2016 ^40^ |  |  |  |  |  |  |  | Some concerns |

1. Bias due to confounding
2. Bias in selection of participants into the study
3. Bias in classification of interventions
4. Bias due to deviations from intended intervention
5. Bias due to missing data
6. Bias in measurement of outcomes
7. Bias in selection of the reported result
8. **Neonatal diagnosis**

**Recommendation D.1b**

| Study | Risk of bias | | | |
| --- | --- | --- | --- | --- |
|  | Patient selection | Index test | Reference standard | Flow and timing |
| Yamamoto 2006 ^41^ |  |  |  |  |
| Leruez-Ville 2017 ^23^ |  |  |  |  |
| Eventov-Friedman 2019 ^42^ |  |  |  |  |
| Exler 2019 ^43^ |  |  |  |  |
| Blazquez_Gamero 2020 ^44^ |  |  |  |  |

**Assessment of risk of bias for saliva neonatal testing using QUADAS-C.**

Low Risk High Risk Unclear Risk

**Recommendation D.1d**

**Assessment of risk of bias for IgM neonatal testing**

| Study | Risk of bias | | | |
| --- | --- | --- | --- | --- |
|  | Patient selection | Index test | Reference standard | Flow and timing |
| Revello 1999 ^45^ |  |  |  |  |
| Bilavsky 2017 ^46^ |  |  |  |  |

Low Risk High Risk Unclear Risk

1. **Neonatal investigation, neonatal treatment and long-term follow-up**

| ***Recommendation E.1c***  **Assessment of risk of studies assessing bias for study assessing the prognosis value of neonatal MRI using Newcastle Ottawa Scale**   \| **Studies** \| **Selection** \| **Comparability** \| **Outcome** \| **Total stars** \| \| --- \| --- \| --- \| --- \| --- \| \| Alarcon, 2013 ^47^ \| **** \| ** \| ** \| 8 \| \| Capretti, 2014 ^48^ \| **** \| ** \| ** \| 8 \| \| Giannattasio A, 2018 ^49^ \| **** \| ** \| ** \| 8 \| \| Blazquez-Gamero, 2019 ^50^ \| **** \| ** \| ** \| 8 \|   ***Recommendation E.1d***  **Assessment of risk of bias of the studies assessing the role of CMV PCR in CSF**   \| **Studies** \| **Selection** \| **Comparability** \| **Outcome** \| **Total stars** \| \| --- \| --- \| --- \| --- \| --- \| \| Goycochea-Valdivia, 2017 ^51^ \| **** \| ** \| *** \| 9 \| \| Czech-Kowalska, 2021 ^52^ \| **** \| ** \| *** \| 9 \|   **Recommendation E.2**  ***Assessment of risk of bias of Randomized controlled trial for 6 months neonatal treatment using risk of bias tool 2*** | | | | | | | |
| --- | --- | --- | --- | --- | --- | --- | --- | --- | --- | --- | --- | --- | --- | --- | --- | --- | --- | --- | --- | --- | --- | --- | --- | --- | --- | --- | --- | --- | --- | --- | --- | --- | --- | --- | --- | --- | --- | --- | --- | --- | --- | --- | --- | --- | --- | --- | --- |
|  | 1 | 2 | 3 | 4 | 5 | 6 | Overall |
| Kimberlin, 2015 ^53^ |  |  |  |  |  |  | Some concerns |
| Kimberlin, 2003 ^54^ |  |  |  |  |  |  | High risk |

1. Bias arising from the randomization process
2. Bias due to deviations from intended interventions (effect of assignment to intervention)
3. Bias due to deviations from intended interventions (effect starting and adhering to intervention)
4. Bias due to missing outcome data
5. Bias in measurement of the outcome
6. Bias in selection of the reported result

**Recommendation E.3**

***Assessment of risk of bias of the study assessing 6 weeks valganciclovir treatment implemented within the first 3 months of life in infected neonates with isolated hearing loss***

| **Studies** | **Selection** | **Comparability** | **Outcome** | **Total stars** |
| --- | --- | --- | --- | --- |
| Vossen, 2023 ^55^ | *** | ** | ** | 7 |

**Recommendation E.4a**

**Assessment of risk of bias of the study assessing the risk of delayed hearing loss according to time**

| **Studies** | **Selection** | **Comparability** | **Outcome** | **Total stars** |
| --- | --- | --- | --- | --- |
| Lanzieri, 2017 ^56^ | *** | * | **** | 8 |

**Recommendation E.4b**

***Assessment of risk of bias of the studies assessing the risk of vestibular dysfunction***

| **Studies** | **Selection** | **Comparability** | **Outcome** | **Total stars** |
| --- | --- | --- | --- | --- |
| Dhondt, 2023 ^57^ | *** |  | **** | 7 |
| Kokkola, 2023 ^58^ | **** | * | **** | 9 |

1. **Supplementary tables**

Table S1: Specificity of IgM screening in pregnancy

| Study | N° of pregnant women screened | Results |
| --- | --- | --- |
| Munro, 2005 ^59^ | 600 | 5% IgM +  20% low avidity/ 80% high avidity |
| De Paschale , 2009 ^60^ | 2,817 | 0.9% IgM +  46% low or intermediate avidity/ 54% high avidity |
| Picone , 2009 ^61^ | 4,297 | 5.7% IgM+  23% low or intermediate/76% high avidity |
| Leruez-Ville, 2013 ^62^ | 4,000 | 4.1% IgM+  41% low or intermediate avidity /59% high avidity |
| Leruez-Ville, 2020 ^63^ | 11,728 | 3.2% IgM+  27% low or intermediate avidity/ 73% high avidity |
| Périllaud-Dubois, 2020 ^64^ | 6,560 | 16.4% positive predictive value of a positive IgM |

Table S2: CMV PCR in blood and urine in primary infection

| Study | Number of primary infection cases | Type of sample | Sensitivity at seroconversion | Duration of CMV PCR positivity from primary infection |
| --- | --- | --- | --- | --- |
| Ziemann, 2010 ^65^ | 13 blood donors | Whole blood, urine | ND | Blood: median: 137 days (0-269)  Urine: median 355 days (0-559 days). |
| Revello, 2014 ^66^ | 597 women | Whole blood | 76% (452/597) |  |
| Berth, 2016 ^18^ | 66 healthy adults | Whole blood | 94% | 50% at D30  14% between D30 and D90  0%: after D90 |
| Sarasini, 2021 ^14^ | 465 women | Whole Blood | 97% | 97%: <D30  80% :D31 to D60; 63%: D61 to D90  50% : D91 to D120  40% : D121-D180; 18% : after D181 |
| Périllaud-Dubois, 2022 ^20^ | 132 women | 74 Whole blood/ 123 sera | In Pi<2 weeks:  100% in whole blood  89% in serum | Whole blood:  78% :D15 to D42  68%: D43 to D90  Serum  65% :D15 to D42  27%: D43 to D90 |
| Fornara C et al, Med Microbiol Immunol, 2022 ^19^ | 33 women | Whole Blood/ urine | 88% in the first D30 | Whole Blood :  88% D30,  32% D180,  0% D180-D730  Urine:  90% D30  95% D60  70% D180  45% D365  12% D730 |

Table S3: Serology and PCR in blood for diagnosis of non-primary infection

|  | Number | % of positive IgM | % of significant rise in IgG level | % of positive DNAemia |
| --- | --- | --- | --- | --- |
| Zalel, 2008 ^67^ | 6 fetal infections following NPI | 0/6 | 0/6 | ND |
| Leruez-Ville, 2017 ^23^ | 20 neonatal infections following NPI | 0/20 (in the first trimester) |  |  |
| Picone, 2017 ^26^ | 9 fetal/neonatal infections following NPI | 2/9 (22%) | 3/9 (33%) | 6/9 (66%) |
| Hadar, 2017 ^22^ | 12 fetal/neonatal infections following NPI | 3/12 (25%) |  |  |
| Puhakka, 2017 ^27^ | 14 fetal/neonatal infections following NPI | 3/14 (21%) |  |  |
| Périllaud-Dubois, 2022 ^25^ | 53 fetal/neonatal infections following NPI, 195 serum | 7/53 (13.2%) | 10/53 (5.7%) | 18/53 (34%) in serum ( mean viral load 46 copies/ml) |
| Zelini, 2022 ^68^ | 250 pre-immune pregnant women | 6/250 (2.4%) |  | 8/250 (3.2%)  (median viral load 57 (1-291) copies/ml) |
| Lilleri, 2023 ^24^ | 17 neonatal infections following NPI | 0/17 in the beginning of pregnancy |  | 4/16 (25%) |

NPI= non primary-infection

Table S4. Practical considerations for pregnant women on preventive treatment with valaciclovir.

| **Treatment Information** | **Comments / Considerations** |
| --- | --- |
| Medication | Valaciclovir tablets of 1000 mg (or 500 mg if 1000 mg tablets are unavailable) |
| Dosing | Valaciclovir 2g four times per day with as close to 6 hours interval as possible  Precaution: water intake of at least 2 liters per day |
| Eligibility | Women with primary infection in the first trimester of pregnancy or in the periconceptional period |
| When to Start | Ideally as soon as possible after the diagnosis of primary infection and before 16 weeks |
| Blood Monitoring | Creatinine clearance and full blood count at baseline then every 2 weeks until discontinuation of treatment. |
| Duration of Treatment | Treatment is discontinued after a negative CMV PCR result in amniotic fluid collected by amniocentesis from 17+0 weeks or between 17 to 18 weeks in women refusing amniocentesis.  In women with positive CMV PCR in amniotic fluid, continuation of valaciclovir 8g / day could be considered after discussion with an expert team |
| Side Effects | Short Term – *whilst on treatment*  *Frequent: Nausea, headaches*  *Rare: thrombocytopenia*, *abnormal renal function*– treatment must be discontinued in case of increase in serum creatinine by ≥0.3 mg/dL (≥26.5 micromol/L) within 48 hours, or increase in serum creatinine to ≥1.5 times baseline, which is known or presumed to have occurred within the prior seven days, or urine volume <0.5 mL/kg/hour for six hours or thrombocytopenia. Renal function and platelets count return to normal off treatment. |
|  | Long Term – potential / unknown consequences of treatment. Use of valaciclovir for CMV primary infection has only been routine since 2019. Valaciclovir for herpes simplex or varicella infection has been used for decades with reassuring data at all stages of pregnancy^97,98^.Animal models showed no evidence of mutagenicity/ teratogenicity when valaciclovir/acyclovir was injected at concentrations equivalent to those used in human clinical practice^99,100^. No such cases reported in treated fetuses so far. |

Table S5: Performance of the PCR in amniotic fluid according to the timing of amniocentesis

| Study | Number of cases | Timing of amniocentesis in weeks | Interval between onset of primary maternal infection and amniocentesis in weeks | Sensitivity of CMV PCR in AF | NPV of CMV PCR in AF | Specificity of CMV PCR in AF | PPV of CMV PCR in AF |
| --- | --- | --- | --- | --- | --- | --- | --- |
| Donner et al, 1994 ^35^ | 11 | <21 |  | 45% |  | 100% |  |
| Revello et al, 1995 ^36^ | 26 | 14-32 |  |  | 81% |  | 100% |
| Liesnard et al, 2000 ^37^ | 237 | <21  >21 | 6.6 when CMV PCR positive in AF  4.5 when CMV PCR negative in AF | 30%  74% |  |  |  |
| Enders et al, 2001^38^ | 242 | <21  >21 |  | 66.7%  96% | 92.7%  97.9% | 100%  100% | 100%  100% |

AF= amniotic fluid

Table S6: Fetal and neonatal neuroimaging scoring systems for cCMV

| **Cannie et al**. ^70^– Fetal MRI | | | Postnatal cUS and MRI  **Alarcon et al**. ^71,72^ | | |
| --- | --- | --- | --- | --- | --- |
| **Score** | **Findings** | **Outcome** | **Score** | **Findings** |  |
| 1 | Normal findings | SNHL: 0%  Neurological impairment:1.6% | 0 | None of the following abnormalities | Normal: 87.5%  Mild disability: 6.3%  Moderate/severe disability: 6.3% |
| 2 | Isolated frontal or parieto-occipital periventricular increased signal intensity on T2 weighted sequence | SNHL: 0%  Neurological impairment:0% | 1 | Single punctate periventricular calcification, lenticulostriate vasculopathy, caudothalamic germinolysis, mild ventriculomegaly* and/or focal/multifocal white matter signal abnormality on MRI | Normal: 85.4%  Mild disability: 8.3%  Moderate/severe disability: 6.3% |
| 3 | Isolated temporal periventricular increased signal intensity on T2 weighted sequence | SNHL: 14.3%  Neurological impairment:0% | 2 | Multiple discrete periventricular calcifications, paraventricular germinolytic cysts, occipital horn septations, moderate/severe ventriculomegaly*, diffuse white matter signal abnormality and/or temporal lobe involvement | Normal: 48.7%  Mild disability: 17.9%  Moderate/severe disability: 35.9% |
| 4 | Cysts and/or septa in the temporal and/or occipital lobe | SNHL: 55%  Neurological impairment:25% |  |  |  |
| 5 | Migrational disorders, cerebellar hypoplasia | SNHL: 66.7%  Neurological impairment:66.7% | 3 | Extensive calcifications, brain atrophy, abnormal gyration, cortical malformation, dysgenesis of the corpus callosum and/or cerebellar hypoplasia | Normal: 3%  Mild disability: 0 %  Moderate/severe disability: 97% |

*Ventriculomegaly is defined as lateral ventricle width ≥7.5 mm on a coronal section at the level of the atria, and classified as mild (7.5-9.9 mm) or moderate/severe (≥10 mm)^73^.

Table S7: Sensitivity, PPV, specificity and NPV of CMV PCR in saliva compared to CMV PCR in urine for diagnosis of cCMV in neonates

| Study | Number of samples | Sensitivity | Specificity | PPV | NPV |
| --- | --- | --- | --- | --- | --- |
| Yamamoto, 2006 (41) | 1923 | 93% | 100% | 100% | 99.9% |
| Ross, 2014 (69) | 80 | 100% |  | 98.7% |  |
| Leruez-Ville, 2017 (23) | 87 |  |  | 58.6% |  |
| Eventov-Friedman, 2019 (42) | 859 | 98.3% | 91.5% | 45.6% | 99.8% |
| Exler, 2019 (43) | 133 | 94.5% | 89.6% | 77.7% | 97.7% |
| Blazquez-Gamero, 2020 (44) | 24 |  |  | 62.5% |  |

Table S8: Symptoms and signs of infants with cCMV.

| **Clinical symptoms/signs on physical examination** |
| --- |
| Intrauterine growth restriction (IUGR; birth weight <-2 SD for GA)* |
| Microcephaly (head circumference (HC) <-2 SD for GA)*† |
| Petechiae or purpura |
| Blueberry muffin rash (intradermal hematopoiesis) |
| Jaundice |
| Hepatomegaly |
| Splenomegaly |
| Abnormal neurological examination (lethargy, hypotonia, seizures, poor suck) |
| **Abnormal laboratory results** |
| Anemia (according to reference hemoglobin and hematocrit values for age and sex) |
| Thrombocytopenia (<100,000 per µL) |
| Leukopenia, isolated neutropenia (<1,000 per µL) |
| Elevated liver enzymes (ALT/AST at least 2 times ULN) |
| Conjugated hyperbilirubinemia (direct bilirubin >2 mg/dL) |
| **Cerebrospinal fluid** (CSF) |
| Abnormal indices, positive CMV DNA |
| **Neuroimaging** |
| The abnormalities can be classified into two types ^47-50,71,74-78^:   1. Inflammatory or destructive changes resulting from the direct effect of the virus or the immune/inflammatory response: lenticulostriate vasculopathy, germinolytic pseudocysts (caudothalamic, temporal, frontal), occipital horn septations, ventriculomegaly, periventricular calcifications, white matter abnormalities (i.e., increased signal intensity on T2-weighted MRI). 2. Brain developmental disruptions: cortical malformations (typically polymicrogyria or poorly developed sulcation), cerebellar hypoplasia. |
| **Hearing evaluation** |
| SNHL (hearing threshold >20 dB, uni- or bilateral) |
| **Ophthalmologic evaluation** |
| Chorioretinitis or scarring |

*ULN*, upper limit of normal

*Use ethnic-specific or multi-ethnic growth charts^79^. cCMV is more likely in symmetric intrauterine growth restriction (IUGR), where weight and HC are proportionally affected. This is in contrast to asymmetric IUGR, where HC is preserved while weight is compromised due to reduced fetal nutrition in the late second or third trimesters.

†Microcephaly in cCMV can be of two types. In *proportional microcephaly*, due to symmetric IUGR, both HC and weight are <-2SD and in proportion to each other. By contrast, *relative microcephaly* (i.e., HC *z* score – weight *z* score <-2) has a high specificity for central nervous system (CNS) involvement and poor neurological outcomes (47,74)

Table S9: Prevalence of cCMV in neonates small for gestational age

|  | Number of neonates | Diagnosis method of cCMV | % cCMV |
| --- | --- | --- | --- |
| Khan, 2000^80^ | 75 (IUGR) | CMV IgM | 0 |
| Van Der Weider, 2011 ^81^ | 112 (median birth weight 1427±640 grams) | PCR in urine | 2% |
| Lorenzoni, 2014 ^82^ |  |  | 3.7% |
| Turner, 2014 ^83^ | 4594  (birth weight 501 to 1500 grams | Not given | 0.39% |
| Espiritu, 2018 ^84^ | 386 (<10°p)  32% (123 tested for CMV) | PCR in Urine | 0 |

Table S10: Neonatal predictors of poor psychomotor development and/or SNHL

| Study | Number of neonates | Number of symptomatic  /asymptomatic | Age at last follow-up in years | Neonatal predictors of poor psychomotor development | Neonatal predictors of SNHL |
| --- | --- | --- | --- | --- | --- |
| Ramsay, 1991 ^85^ | 65 | 65/65 (100%) | 3 to 4 | Microcephaly |  |
| Conboy, 1987 ^86^ | 32 | 32/32 (100%) | 6.4 | Microcephaly |  |
| Noyola, 2001 ^74^ | 41 | 41/41 (100%) | 5.6 | Microcephaly  Abnormal CT scan |  |
| Alarcon, 2013 ^47^ | 26 | 26/26 (100%) | 8.7 | Abnormal neuro-imaging |  |
| Capretti, 2014 ^48^ | 40 | 20/40 (25%) | >2 | Abnormal ultrasound and abnormal MRI |  |
| Giannattasio A, 2018 ^49^ | 170 | 112/170 (66%) |  | Severity of neuroimaging score |  |
| Lanzieri, 2017 ^56^ | 76 | 76/75 (100%) | 13 | Microcephaly  Tissue destruction at CT scan |  |
| Blazquez-Gamero, 2019^50^ | 107 | 77/107(72%) | 1 | Abnormal MRI |  |
| Rivera, 2002 ^87^ | 190 | 190/190 (100%) | 5.7 |  | Petechia  IUGR |
| De Cuyper, 2023 ^88^ | 1033 | 416/1033 (40%) | Birth |  | Petechial  Periventricular cyst at MRI |

Table S11: Predictive value of neonatal CMV blood viral load (BVL)

| Study | Number of  neonates | BVL higher in symptomatic | BVL higher in  Case with sequelae (HL or other) | Threshold BVL associated with the absence of sequelae | Threshold BVL associated with sequelae |
| --- | --- | --- | --- | --- | --- |
| Boppana, 2005 ^89^ | 75 | yes | yes | <10,000 copies/ mL  NPV=100% | None |
| Lanari, 2006 ^90^ | 44 | yes | yes | <1,000copies/PMNLs | > 10,000/PMNLs |
| Walter, 2008 ^91^ | 43 |  | yes |  |  |
| Ross, 2009 ^92^ | 135 |  | no | <3500 genome/mL  NPV=94% | None |
| Forner, 2015 ^93^ | 33 |  | yes |  | >12,000 copies/ml  PPV=50% |
| Smiljkovic, 2020 ^94^ | 47 | yes | yes |  | >18,770 copies/mL  PPV=75%  >100,000 copies/ml  PPV=100% |
| Marsico, 2019 ^95^ | 47 | yes | yes | None | None |
| Fourgeaud, 2023 ^96^ | 256 | yes | yes | <1000 copies/ml  NPV=100% | None |

Table S12– Practical considerations for infants on treatment with valganciclovir or ganciclovir for cCMV

| **Treatment Information** | **Comments / Considerations** |
| --- | --- |
| Medication | Valganciclovir oral liquid is the treatment of choice.  Ganciclovir intravenously may be used for infants unable to take enteral medication or in very severe cases |
| Dosing | Valganciclovir liquid orally – 16 mg / Kg per dose, twice daily  Ganciclovir intravenously – 6mg / Kg per dose, twice daily (minimize use to reduce risk of side effects, change to oral valganciclovir as soon as possible, avoid use for more than 6 weeks) |
| Gestational Eligibility | Infants weight > 1.8 kgs and /or 32 weeks’ gestation at delivery  *If considering treatment in smaller or more premature infants seek expert advice, drug level monitoring should be undertaken, if available.* |
| When to Start | Ideally as soon as possible after birth, and before 4 weeks of age.  *If there is a delay in diagnosis, then may still be started up to 12 weeks of age.*  *In selected cases, if considering starting treatment after 12 weeks of age, this should be discussed with an expert* |
| Blood Monitoring | Full Blood Count, liver function tests at: baseline, 2 weeks, 4 weeks, then once 3 weeks after completion of treatment.  CMV DNA blood viral load at: at least at baseline, some clinics also do viral load with other routine blood tests to monitor treatment efficacy. |
| Duration of Treatment | Any CNS involvement (including hearing loss, chorioretinitis, clinical symptoms, or abnormal brain imaging) – 6 months  No CNS involvement (e.g. symptomatic isolated hepatitis or isolated thrombocytopenia) – 6 weeks or more, depending on response. |
| Side Effects | Short Term – *whilst on treatment*  *Bone marrow suppression* – most commonly neutropenia, rarely anemia or thrombocytopenia  Data from CCMVNET Registry: severe neutropenia on treatment (<0.5) – 18.4% of all infants; IV Ganciclovir for>14d, prematurity (GA <36 weeks); and lower pre-treatment ANC were all strongly associated with severe neutropenia.  Neutrophil count:  > 1.0 Normal follow-up  > 0.8 –1.0, test in 2 weeks – continue treatment, advise parents what to do if becomes febrile / unwell.  0.5 - 0.8, test in 1 week – continue treatment, advise parents what to do if becomes febrile / unwell.  <0.5 – stop treatment, test in 1-2 weeks, only re-start if >0.8-1.0, advise parents what to do if becomes febrile / unwell.  Although no consensus, granulocyte colony stimulating factor (G-CSF) treatment may be used in infants with persistent neutropenia, when antiviral treatment is considered of paramount importance, seek expert advice.  *Abnormal Liver Function Tests* – mild rise in ALT / AST may occur, usually as treatment progresses, rarely more than 2-4 times ULN, usually returns to normal off treatment. |
|  | Long Term – potential / unknown consequences of treatment. Use of ganciclovir / valganciclovir for cCMV infection has only been routine since 2003. Animal studies suggest a possible risk of mutagenicity / teratogenicity. No such cases reported in treated infants so far. |
| Parent Information | Parents should be informed about the clinical trial data on Valganciclovir and / or Ganciclovir, both in terms of efficacy and side effects. Open discussion is important in joint decision making about starting treatment, as there is a delicate balance of benefits versus risks.  Parents should be informed of who to contact, and what to do if their infant becomes unwell with possible febrile neutropenia.  Parents should be given access to websites and information leaflets (see example attached), with contact details to discuss their concerns. |

Figure S1: Algorithm for interpretation of CMV serology in the first trimester of pregnancy

CMV IgG & IgM as early as possible (within first trimester)

IgG negative

IgM negative *

IgG positive**

IgM negative

IgG positive

IgM positive

IgG negative

IgM positive

PCR in blood immediatly

and

Serology in 10 to 15 days

Low avidity

IgG avidity testing****

High probability of PI

in the first trimester

or periconceptional

period

High avidity***

Exclude PI

in first trimester and

periconceptional period

with high probability

Intermediate

avidity

Cannot exclude a PI

in the first trimester

or periconceptional

period

Retest with another avidity assay

Intermediate

or low avidity

High avidity

Retest every 4 weeks up to

14 to 16 weeks

Negative PCR

No IgG seroconversion

Exclude

PI

Positive PCR

IgG seroconversion

Confirm

PI

Figure S1 legends:

* Use a sensitive IgM assay

** Sera with weakly positive IgG results (< twice the threshold value) should be retested with a second assay or send to a reference laboratory. Sera that are positive with both assays can be declared positive; those with discordant results should be considered equivocal and declared negative.

*** Caution: in acute infection with low IgG levels and positive IgM: the avidity may be falsely high (described with some assays). Such cases may be suspected when low IgG levels are associated with high IgM level, evocative of a recent PI but contrasting with high avidity. Testing a subsequent serum shows rise in IgG, decrease of IgM and the PCR in blood is positive.

****Caution: in sera with low IgG levels (< twice the threshold level), the avidity may be falsely low or intermediate. Such cases may be suspected when low IgG are associated with low IgM and low avidity. Testing a subsequent serum shows no change in IgG and IgM kinetic and CMV PCR is negative in blood.

**Bibliography**

1. Adler SP, Finney JW, Manganello AM, Best AM. Prevention of child-to-mother transmission of cytomegalovirus among pregnant women. J Pediatr. oct 2004;145(4):485‑91.

2. Vauloup-Fellous C, Picone O, Cordier AG, Parent-du-Châtelet I, Senat MV, Frydman R, et al. Does hygiene counseling have an impact on the rate of CMV primary infection during pregnancy? Results of a 3-year prospective study in a French hospital. J Clin Virol Off Publ Pan Am Soc Clin Virol. déc 2009;46 Suppl 4:S49-53.

3. Revello MG, Tibaldi C, Masuelli G, Frisina V, Sacchi A, Furione M, et al. Prevention of Primary Cytomegalovirus Infection in Pregnancy. EBioMedicine. sept 2015;2(9):1205‑10.

4. Leruez-Ville M, Guilleminot T, Stirnemann J, Salomon LJ, Spaggiari E, Faure-Bardon V, et al. Quantifying the Burden of Congenital Cytomegalovirus Infection With Long-term Sequelae in Subsequent Pregnancies of Women Seronegative at Their First Pregnancy. Clin Infect Dis Off Publ Infect Dis Soc Am. 23 oct 2020;71(7):1598‑603.

5. Binda S, Pellegrinelli L, Terraneo M, Caserini A, Primache V, Bubba L, et al. What people know about congenital CMV: an analysis of a large heterogeneous population through a web-based survey. BMC Infect Dis. 26 sept 2016;16(1):513.

6. Fowler KB, Stagno S, Pass RF. Interval between births and risk of congenital cytomegalovirus infection. Clin Infect Dis Off Publ Infect Dis Soc Am. 1 avr 2004;38(7):1035‑7.

7. Fellah T, Sibiude J, Vauloup-Fellous C, Cordier AG, Guitton S, Grangeot-Keros L, et al. Evolution of awareness and knowledge of congenital cytomegalovirus infection among health care providers in France between 2011 and 2018. J Clin Virol Off Publ Pan Am Soc Clin Virol. août 2020;129:104335.

8. Castillo K, Hawkins-Villarreal A, Valdés-Bango M, Guirado L, Scazzocchio E, Porta O, et al. Congenital Cytomegalovirus Awareness and Knowledge among Health Professionals and Pregnant Women: An Action towards Prevention. Fetal Diagn Ther. 2022;49(5‑6):265‑72.

9. Carlier P, Harika N, Bailly R, Vranken G. Laboratory evaluation of the new Access ® cytomegalovirus immunoglobulin IgM and IgG assays. J Clin Virol Off Publ Pan Am Soc Clin Virol. nov 2010;49(3):192‑7.

10. Chiereghin A, Pavia C, Gabrielli L, Piccirilli G, Squarzoni D, Turello G, et al. Clinical evaluation of the new Roche platform of serological and molecular cytomegalovirus-specific assays in the diagnosis and prognosis of congenital cytomegalovirus infection. J Virol Methods. oct 2017;248:250‑4.

11. Delforge ML, Desomberg L, Montesinos I. Evaluation of the new LIAISON(®) CMV IgG, IgM and IgG Avidity II assays. J Clin Virol Off Publ Pan Am Soc Clin Virol. nov 2015;72:42‑5.

12. Genco F, Sarasini A, Parea M, Prestia M, Scudeller L, Meroni V. Comparison of the LIAISON®XL and ARCHITECT IgG, IgM, and IgG avidity assays for the diagnosis of Toxoplasma, cytomegalovirus, and rubella virus infections. New Microbiol. avr 2019;42(2):88‑93.

13. Revello MG, Vauloup-Fellous C, Grangeot-Keros L, van Helden J, Dickstein Y, Lipkin I, et al. Clinical evaluation of new automated cytomegalovirus IgM and IgG assays for the Elecsys(®) analyser platform. Eur J Clin Microbiol Infect Dis Off Publ Eur Soc Clin Microbiol. déc 2012;31(12):3331‑9.

14. Sarasini A, Arossa A, Zavattoni M, Fornara C, Lilleri D, Spinillo A, et al. Pitfalls in the Serological Diagnosis of Primary Human Cytomegalovirus Infection in Pregnancy Due to Different Kinetics of IgM Clearance and IgG Avidity Index Maturation. Diagn Basel Switz. 26 févr 2021;11(3):396.

15. Lagrou K, Bodeus M, Van Ranst M, Goubau P. Evaluation of the new architect cytomegalovirus immunoglobulin M (IgM), IgG, and IgG avidity assays. J Clin Microbiol. juin 2009;47(6):1695‑9.

16. Vauloup-Fellous C, Berth M, Heskia F, Dugua JM, Grangeot-Keros L. Re-evaluation of the VIDAS(®) cytomegalovirus (CMV) IgG avidity assay: determination of new cut-off values based on the study of kinetics of CMV-IgG maturation. J Clin Virol Off Publ Pan Am Soc Clin Virol. févr 2013;56(2):118‑23.

17. Vauloup-Fellous C, Lazzarotto T, Revello MG, Grangeot-Keros L. Clinical evaluation of the Roche Elecsys CMV IgG Avidity assay. Eur J Clin Microbiol Infect Dis Off Publ Eur Soc Clin Microbiol. août 2014;33(8):1365‑9.

18. Berth M, Benoy I, Christensen N. Evaluation of a standardised real-time PCR based DNA-detection method (Realstar®) in whole blood for the diagnosis of primary human cytomegalovirus (CMV) infections in immunocompetent patients. Eur J Clin Microbiol Infect Dis Off Publ Eur Soc Clin Microbiol. févr 2016;35(2):245‑9.

19. Fornara C, Zavaglio F, Furione M, Sarasini A, d’Angelo P, Arossa A, et al. Human cytomegalovirus (HCMV) long-term shedding and HCMV-specific immune response in pregnant women with primary HCMV infection. Med Microbiol Immunol (Berl). déc 2022;211(5‑6):249‑60.

20. Périllaud-Dubois C, Bouthry E, Mouna L, Pirin C, Vieux-Combe C, Picone O, et al. Contribution of Serum Cytomegalovirus PCR to Diagnosis of Early CMV Primary Infection in Pregnant Women. Viruses. 28 sept 2022;14(10):2137.

21. Revello MG, Lilleri D, Zavattoni M, Stronati M, Bollani L, Middeldorp JM, et al. Human cytomegalovirus immediate-early messenger RNA in blood of pregnant women with primary infection and of congenitally infected newborns. J Infect Dis. 15 oct 2001;184(8):1078‑81.

22. Hadar E, Dorfman E, Bardin R, Gabbay-Benziv R, Amir J, Pardo J. Symptomatic congenital cytomegalovirus disease following non-primary maternal infection: a retrospective cohort study. BMC Infect Dis. 5 janv 2017;17(1):31.

23. Leruez-Ville M, Magny JF, Couderc S, Pichon C, Parodi M, Bussières L, et al. Risk Factors for Congenital Cytomegalovirus Infection Following Primary and Nonprimary Maternal Infection: A Prospective Neonatal Screening Study Using Polymerase Chain Reaction in Saliva. Clin Infect Dis Off Publ Infect Dis Soc Am. 1 août 2017;65(3):398‑404.

24. Lilleri D, Tassis B, Pugni L, Ronchi A, Pietrasanta C, Spinillo A, et al. Prevalence, Outcome, and Prevention of Congenital Cytomegalovirus Infection in Neonates Born to Women With Preconception Immunity (CHILd Study). Clin Infect Dis Off Publ Infect Dis Soc Am. 8 févr 2023;76(3):513‑20.

25. Périllaud-Dubois C, Letamendia E, Bouthry E, Rafek R, Thouard I, Vieux-Combe C, et al. Cytomegalovirus Specific Serological and Molecular Markers in a Series of Pregnant Women With Cytomegalovirus Non Primary Infection. Viruses. 31 oct 2022;14(11):2425.

26. Picone O, Grangeot-Keros L, Senat M, Fuchs F, Bouthry E, Ayoubi J, et al. Cytomegalovirus non-primary infection during pregnancy. Can serology help with diagnosis? J Matern-Fetal Neonatal Med Off J Eur Assoc Perinat Med Fed Asia Ocean Perinat Soc Int Soc Perinat Obstet. janv 2017;30(2):224‑7.

27. Puhakka L, Renko M, Helminen M, Peltola V, Heiskanen-Kosma T, Lappalainen M, et al. Primary versus non-primary maternal cytomegalovirus infection as a cause of symptomatic congenital infection - register-based study from Finland. Infect Dis Lond Engl. juin 2017;49(6):445‑53.

28. Shahar-Nissan K, Pardo J, Peled O, Krause I, Bilavsky E, Wiznitzer A, et al. Valaciclovir to prevent vertical transmission of cytomegalovirus after maternal primary infection during pregnancy: a randomised, double-blind, placebo-controlled trial. Lancet Lond Engl. 12 sept 2020;396(10253):779‑85.

29. Faure-Bardon V, Fourgeaud J, Stirnemann J, Leruez-Ville M, Ville Y. Secondary prevention of congenital cytomegalovirus infection with valacyclovir following maternal primary infection in early pregnancy. Ultrasound Obstet Gynecol Off J Int Soc Ultrasound Obstet Gynecol. oct 2021;58(4):576‑81.

30. Egloff C, Sibiude J, Vauloup-Fellous C, Benachi A, Bouthry E, Biquard F, et al. New data on efficacy of valacyclovir in secondary prevention of maternal-fetal transmission of cytomegalovirus. Ultrasound Obstet Gynecol Off J Int Soc Ultrasound Obstet Gynecol. janv 2023;61(1):59‑66.

31. Ville Y, Leruez-Ville M. Renal toxicity of high-dosage valacyclovir for secondary prevention of congenital cytomegalovirus infection: a dose regimen-related issue. Ultrasound Obstet Gynecol Off J Int Soc Ultrasound Obstet Gynecol. oct 2021;58(4):637‑8.

32. Revello MG, Lazzarotto T, Guerra B, Spinillo A, Ferrazzi E, Kustermann A, et al. A randomized trial of hyperimmune globulin to prevent congenital cytomegalovirus. N Engl J Med. 3 avr 2014;370(14):1316‑26.

33. Hughes BL, Clifton RG, Rouse DJ, Saade GR, Dinsmoor MJ, Reddy UM, et al. A Trial of Hyperimmune Globulin to Prevent Congenital Cytomegalovirus Infection. N Engl J Med. 29 juill 2021;385(5):436‑44.

34. Kagan KO, Enders M, Schampera MS, Baeumel E, Hoopmann M, Geipel A, et al. Prevention of maternal-fetal transmission of cytomegalovirus after primary maternal infection in the first trimester by biweekly hyperimmunoglobulin administration. Ultrasound Obstet Gynecol Off J Int Soc Ultrasound Obstet Gynecol. mars 2019;53(3):383‑9.

35. Donner C, Liesnard C, Brancart F, Rodesch F. Accuracy of amniotic fluid testing before 21 weeks’ gestation in prenatal diagnosis of congenital cytomegalovirus infection. Prenat Diagn. nov 1994;14(11):1055‑9.

36. Revello MG, Baldanti F, Furione M, Sarasini A, Percivalle E, Zavattoni M, et al. Polymerase chain reaction for prenatal diagnosis of congenital human cytomegalovirus infection. J Med Virol. déc 1995;47(4):462‑6.

37. Liesnard C, Donner C, Brancart F, Gosselin F, Delforge ML, Rodesch F. Prenatal diagnosis of congenital cytomegalovirus infection: prospective study of 237 pregnancies at risk. Obstet Gynecol. juin 2000;95(6 Pt 1):881‑8.

38. Enders G, Bäder U, Lindemann L, Schalasta G, Daiminger A. Prenatal diagnosis of congenital cytomegalovirus infection in 189 pregnancies with known outcome. Prenat Diagn. mai 2001;21(5):362‑77.

39. Enders M, Daiminger A, Exler S, Enders G. Amniocentesis for prenatal diagnosis of cytomegalovirus infection: challenging the 21 weeks’ threshold. Prenat Diagn. sept 2017;37(9):940‑2.

40. Leruez-Ville M, Stirnemann J, Sellier Y, Guilleminot T, Dejean A, Magny JF, et al. Feasibility of predicting the outcome of fetal infection with cytomegalovirus at the time of prenatal diagnosis. Am J Obstet Gynecol. sept 2016;215(3):342.e1-9.

41. Yamamoto AY, Mussi-Pinhata MM, Marin LJ, Brito RM, Oliveira PFC, Coelho TB. Is saliva as reliable as urine for detection of cytomegalovirus DNA for neonatal screening of congenital CMV infection? J Clin Virol Off Publ Pan Am Soc Clin Virol. juill 2006;36(3):228‑30.

42. Eventov-Friedman S, Manor H, Bar-Oz B, Averbuch D, Caplan O, Lifshitz A, et al. Saliva Real-Time Polymerase Chain Reaction for Targeted Screening of Congenital Cytomegalovirus Infection. J Infect Dis. 22 oct 2019;220(11):1790‑6.

43. Exler S, Daiminger A, Grothe M, Schalasta G, Enders G, Enders M. Primary cytomegalovirus (CMV) infection in pregnancy: Diagnostic value of CMV PCR in saliva compared to urine at birth. J Clin Virol Off Publ Pan Am Soc Clin Virol. août 2019;117:33‑6.

44. Blázquez-Gamero D, Soriano-Ramos M, Vicente M, Pallás-Alonso CR, Pérez-Rivilla A, García-Álvarez M, et al. Prevalence and Clinical Manifestations of Congenital Cytomegalovirus Infection in a Screening Program in Madrid (PICCSA Study). Pediatr Infect Dis J. nov 2020;39(11):1050‑6.

45. Revello MG, Zavattoni M, Baldanti F, Sarasini A, Paolucci S, Gerna G. Diagnostic and prognostic value of human cytomegalovirus load and IgM antibody in blood of congenitally infected newborns. J Clin Virol Off Publ Pan Am Soc Clin Virol. sept 1999;14(1):57‑66.

46. Bilavsky E, Watad S, Levy I, Linder N, Pardo J, Ben-Zvi H, et al. Positive IgM in Congenital CMV Infection. Clin Pediatr (Phila). avr 2017;56(4):371‑5.

47. Alarcon A, Martinez-Biarge M, Cabañas F, Hernanz A, Quero J, Garcia-Alix A. Clinical, Biochemical, and Neuroimaging Findings Predict Long-Term Neurodevelopmental Outcome in Symptomatic Congenital Cytomegalovirus Infection. J Pediatr. sept 2013;163(3):828-834.e1.

48. Capretti MG, Lanari M, Tani G, Ancora G, Sciutti R, Marsico C, et al. Role of cerebral ultrasound and magnetic resonance imaging in newborns with congenital cytomegalovirus infection. Brain Dev. mars 2014;36(3):203‑11.

49. Giannattasio A, Bruzzese D, Di Costanzo P, Capone E, Romano A, D’Amico A, et al. Neuroimaging Profiles and Neurodevelopmental Outcome in Infants With Congenital Cytomegalovirus Infection. Pediatr Infect Dis J. oct 2018;37(10):1028‑33.

50. Blázquez-Gamero D, Soriano-Ramos M, Martínez de Aragón A, Baquero-Artigao F, Frick MA, Noguera-Julian A, et al. Role of Magnetic Resonance Imaging and Cranial Ultrasonography in Congenital Cytomegalovirus Infection. Pediatr Infect Dis J. nov 2019;38(11):1131‑7.

51. Goycochea-Valdivia WA, Baquero-Artigao F, Del Rosal T, Frick MA, Rojo P, Echeverría MJ, et al. Cytomegalovirus DNA Detection by Polymerase Chain Reaction in Cerebrospinal Fluid of Infants With Congenital Infection: Associations With Clinical Evaluation at Birth and Implications for Follow-up. Clin Infect Dis Off Publ Infect Dis Soc Am. 15 mai 2017;64(10):1335‑42.

52. Czech-Kowalska J, Jedlińska-Pijanowska D, Kasztelewicz B, Kłodzińska M, Pietrzyk A, Sarkaria E, et al. The Limitations of Cytomegalovirus DNA Detection in Cerebrospinal Fluid of Newborn Infants With Congenital CMV Infection: A Tertiary Care Neonatal Center Experience. Pediatr Infect Dis J. 1 sept 2021;40(9):838‑45.

53. Kimberlin DW, Jester PM, Sánchez PJ, Ahmed A, Arav-Boger R, Michaels MG, et al. Valganciclovir for symptomatic congenital cytomegalovirus disease. N Engl J Med. 5 mars 2015;372(10):933‑43.

54. Kimberlin DW, Lin CY, Sánchez PJ, Demmler GJ, Dankner W, Shelton M, et al. Effect of ganciclovir therapy on hearing in symptomatic congenital cytomegalovirus disease involving the central nervous system: a randomized, controlled trial. J Pediatr. juill 2003;143(1):16‑25.

55. Chung K. The Concert trial: treatment of infants with congenital cytomegalovirus and isolated hearing loss. 33rd ECCMID; 2023 avr 16; Copenhagen.

56. Lanzieri TM, Leung J, Caviness AC, Chung W, Flores M, Blum P, et al. Long-term outcomes of children with symptomatic congenital cytomegalovirus disease. J Perinatol Off J Calif Perinat Assoc. juill 2017;37(7):875‑80.

57. Dhondt C, Maes L, Van Acker E, Martens S, Vanaudenaerde S, Rombaut L, et al. Vestibular Follow-up Program for Congenital Cytomegalovirus Based on 6 Years of Longitudinal Data Collection. Ear Hear. 1 mai 2023;

58. Kokkola E, Niemensivu R, Lappalainen M, Palomäki M, Nieminen T, Boppana S, et al. Long-term outcome of vestibular function and hearing in children with congenital cytomegalovirus infection: a prospective cohort study. Eur Arch Oto-Rhino-Laryngol Off J Eur Fed Oto-Rhino-Laryngol Soc EUFOS Affil Ger Soc Oto-Rhino-Laryngol - Head Neck Surg. juill 2023;280(7):3141‑7.

59. Munro SC, Hall B, Whybin LR, Leader L, Robertson P, Maine GT, et al. Diagnosis of and screening for cytomegalovirus infection in pregnant women. J Clin Microbiol. sept 2005;43(9):4713‑8.

60. De Paschale M, Agrappi C, Manco MT, Paganini A, Clerici P. Incidence and risk of cytomegalovirus infection during pregnancy in an urban area of Northern Italy. Infect Dis Obstet Gynecol. 2009;2009:206505.

61. Picone O, Vauloup-Fellous C, Cordier AG, Parent Du Châtelet I, Senat MV, Frydman R, et al. A 2-year study on cytomegalovirus infection during pregnancy in a French hospital. BJOG Int J Obstet Gynaecol. mai 2009;116(6):818‑23.

62. Leruez-Ville M, Sellier Y, Salomon LJ, Stirnemann JJ, Jacquemard F, Ville Y. Prediction of fetal infection in cases with cytomegalovirus immunoglobulin M in the first trimester of pregnancy: a retrospective cohort. Clin Infect Dis Off Publ Infect Dis Soc Am. mai 2013;56(10):1428‑35.

63. Leruez-Ville M, Foulon I, Pass R, Ville Y. Cytomegalovirus infection during pregnancy: state of the science. Am J Obstet Gynecol. sept 2020;223(3):330‑49.

64. Périllaud-Dubois C, Bouthry E, Jadoui A, Leng AL, Roque-Afonso AM, Vauloup-Fellous C. Positive predictive values of CMV-IgM and importance of CMV-IgG avidity testing in detecting primary infection in three different clinical settings. A French retrospective cohort study. J Clin Virol Off Publ Pan Am Soc Clin Virol. nov 2020;132:104641.

65. Ziemann M, Unmack A, Steppat D, Juhl D, Görg S, Hennig H. The natural course of primary cytomegalovirus infection in blood donors. Vox Sang. 1 juill 2010;99(1):24‑33.

66. Revello MG, Furione M, Rognoni V, Arossa A, Gerna G. Cytomegalovirus DNAemia in pregnant women. J Clin Virol Off Publ Pan Am Soc Clin Virol. déc 2014;61(4):590‑2.

67. Zalel Y, Gilboa Y, Berkenshtat M, Yoeli R, Auslander R, Achiron R, et al. Secondary cytomegalovirus infection can cause severe fetal sequelae despite maternal preconceptional immunity. Ultrasound Obstet Gynecol Off J Int Soc Ultrasound Obstet Gynecol. avr 2008;31(4):417‑20.

68. Zelini P, d’Angelo P, De Cicco M, Achille C, Sarasini A, Fiorina L, et al. Human cytomegalovirus non-primary infection during pregnancy: antibody response, risk factors and newborn outcome. Clin Microbiol Infect Off Publ Eur Soc Clin Microbiol Infect Dis. oct 2022;28(10):1375‑81.

69. Ross SA, Ahmed A, Palmer AL, Michaels MG, Sánchez PJ, Bernstein DI, et al. Detection of congenital cytomegalovirus infection by real-time polymerase chain reaction analysis of saliva or urine specimens. J Infect Dis. 1 nov 2014;210(9):1415‑8.

70. Cannie MM, Devlieger R, Leyder M, Claus F, Leus A, De Catte L, et al. Congenital cytomegalovirus infection: contribution and best timing of prenatal MR imaging. Eur Radiol. oct 2016;26(10):3760‑9.

71. Alarcon A, Martinez-Biarge M, Cabañas F, Quero J, García-Alix A. A Prognostic Neonatal Neuroimaging Scale for Symptomatic Congenital Cytomegalovirus Infection. Neonatology. 2016;110(4):277‑85.

72. Alarcón A, de Vries Ls, Parodi A, Arnáez J, Cabañas F, Steggerda Sj, et al. Neuroimaging in infants with congenital cytomegalovirus infection and its correlation with outcome: emphasis on white matter abnormalities. Arch Dis Child Fetal Neonatal Ed [Internet]. 22 sept 2023 [cité 21 nov 2023]; Disponible sur: https://pubmed.ncbi.nlm.nih.gov/37739774/

73. Kidokoro H, Neil JJ, Inder TE. New MR imaging assessment tool to define brain abnormalities in very preterm infants at term. AJNR Am J Neuroradiol. déc 2013;34(11):2208‑14.

74. Noyola DE, Demmler GJ, Nelson CT, Griesser C, Williamson WD, Atkins JT, et al. Early predictors of neurodevelopmental outcome in symptomatic congenital cytomegalovirus infection. J Pediatr. mars 2001;138(3):325‑31.

75. The Spectrum of Cranial Ultrasound and Magnetic Resonance Imaging Abnormalities in Congenital Cytomegalovirus Infection. Neuropediatrics. avr 2004;35(2):113‑9.

76. Oosterom N, Nijman J, Gunkel J, Wolfs TFW, Groenendaal F, Verboon-Maciolek MA, et al. Neuro-Imaging Findings in Infants with Congenital Cytomegalovirus Infection: Relation to Trimester of Infection. Neonatology. 10 mars 2015;107(4):289‑96.

77. Vande Walle C, Keymeulen A, Schiettecatte E, Acke F, Dhooge I, Smets K, et al. Brain MRI findings in newborns with congenital cytomegalovirus infection: results from a large cohort study. Eur Radiol. oct 2021;31(10):8001‑10.

78. Keymeulen A, De Leenheer E, Casaer A, Cossey V, Herregods N, Laroche S, et al. Cranial ultrasound and MRI: complementary or not in the diagnostic assessment of children with congenital CMV infection? Eur J Pediatr. mars 2022;181(3):911‑20.

79. Villar J, Ismail LC, Victora CG, Ohuma EO, Bertino E, Altman DG, et al. International standards for newborn weight, length, and head circumference by gestational age and sex: the Newborn Cross-Sectional Study of the INTERGROWTH-21st Project. The Lancet. sept 2014;384(9946):857‑68.

80. Khan NA, Kazzi SN. Yield and costs of screening growth-retarded infants for torch infections. Am J Perinatol. 2000;17(3):131‑5.

81. van der Weiden S, de Jong EP, Te Pas AB, Middeldorp JM, Vossen ACTM, Rijken M, et al. Is routine TORCH screening and urine CMV culture warranted in small for gestational age neonates? Early Hum Dev. févr 2011;87(2):103‑7.

82. Lorenzoni F, Lunardi S, Liumbruno A, Ferri G, Madrigali V, Fiorentini E, et al. Neonatal screening for congenital cytomegalovirus infection in preterm and small for gestational age infants. J Matern-Fetal Neonatal Med Off J Eur Assoc Perinat Med Fed Asia Ocean Perinat Soc Int Soc Perinat Obstet. oct 2014;27(15):1589‑93.

83. Turner KM, Lee HC, Boppana SB, Carlo WA, Randolph DA. Incidence and impact of CMV infection in very low birth weight infants. Pediatrics. mars 2014;133(3):e609-615.

84. Espiritu MM, Bailey S, Wachtel EV, Mally PV. Utility of routine urine CMV PCR and total serum IgM testing of small for gestational age infants: a single center review. J Perinat Med. 26 janv 2018;46(1):81‑6.

85. Ramsay ME, Miller E, Peckham CS. Outcome of confirmed symptomatic congenital cytomegalovirus infection. Arch Dis Child. sept 1991;66(9):1068‑9.

86. Conboy TJ, Pass RF, Stagno S, Alford CA, Myers GJ, Britt WJ, et al. Early clinical manifestations and intellectual outcome in children with symptomatic congenital cytomegalovirus infection. J Pediatr. sept 1987;111(3):343‑8.

87. Rivera LB, Boppana SB, Fowler KB, Britt WJ, Stagno S, Pass RF. Predictors of hearing loss in children with symptomatic congenital cytomegalovirus infection. Pediatrics. oct 2002;110(4):762‑7.

88. De Cuyper E, Acke F, Keymeulen A, De Leenheer EMR, Van Hoecke H, Padalko E, et al. Risk Factors for Hearing Loss at Birth in Newborns With Congenital Cytomegalovirus Infection. JAMA Otolaryngol-- Head Neck Surg. 1 févr 2023;149(2):122‑30.

89. Boppana SB, Fowler KB, Pass RF, Rivera LB, Bradford RD, Lakeman FD, et al. Congenital cytomegalovirus infection: association between virus burden in infancy and hearing loss. J Pediatr. juin 2005;146(6):817‑23.

90. Lanari M, Lazzarotto T, Venturi V, Papa I, Gabrielli L, Guerra B, et al. Neonatal cytomegalovirus blood load and risk of sequelae in symptomatic and asymptomatic congenitally infected newborns. Pediatrics. janv 2006;117(1):e76-83.

91. Walter S, Atkinson C, Sharland M, Rice P, Raglan E, Emery VC, et al. Congenital cytomegalovirus: association between dried blood spot viral load and hearing loss. Arch Dis Child Fetal Neonatal Ed. juill 2008;93(4):F280-285.

92. Ross SA, Novak Z, Fowler KB, Arora N, Britt WJ, Boppana SB. Cytomegalovirus blood viral load and hearing loss in young children with congenital infection. Pediatr Infect Dis J. juill 2009;28(7):588‑92.

93. Forner G, Abate D, Mengoli C, Palù G, Gussetti N. High Cytomegalovirus (CMV) DNAemia Predicts CMV Sequelae in Asymptomatic Congenitally Infected Newborns Born to Women With Primary Infection During Pregnancy. J Infect Dis. 1 juill 2015;212(1):67‑71.

94. Smiljkovic M, Le Meur JB, Malette B, Boucoiran I, Minsart AF, Lamarre V, et al. Blood viral load in the diagnostic workup of congenital cytomegalovirus infection. J Clin Virol. 1 janv 2020;122:104231.

95. Marsico C, Aban I, Kuo H, James SH, Sanchez PJ, Ahmed A, et al. Blood Viral Load in Symptomatic Congenital Cytomegalovirus Infection. J Infect Dis. 16 avr 2019;219(9):1398‑406.

96. Fourgeaud J, Magny JF, Couderc S, Garcia P, Maillotte AM, Benard M, et al. Clinical Value of Serial Quantitative Analysis of Cytomegalovirus DNA in Blood and Saliva Over the First 24 Months of Life in Congenital Infection: The French Cymepedia Cohort. J Pediatr. févr 2023;253:197-204.e5.

97. Stone KM, Reiff-Eldridge R, White AD, Cordero JF, Brown Z, Alexander ER, et al. Pregnancy outcomes following systemic prenatal acyclovir exposure: Conclusions from the international acyclovir pregnancy registry, 1984-1999. Birt Defects Res A Clin Mol Teratol. 2004 Apr;70(4):201–7.

98. Pasternak B, Hviid A. Use of acyclovir, valacyclovir, and famciclovir in the first trimester of pregnancy and the risk of birth defects. JAMA. 2010 Aug 25;304(8):859–66.

99. Chahoud I, Stahlmann R, Bochert G, Dillmann I, Neubert D. Gross-structural defects in rats after acyclovir application on day 10 of gestation. Arch Toxicol. 1988 Aug;62(1):8–1

100. Nihi F, Moreira D, Santos Lourenço AC, Gomes C, Araujo SL, Zaia RM, et al. Testicular effects following in utero exposure to the antivirals acyclovir and ganciclovir in rats. Toxicol Sci Off J Soc Toxicol. 2014 May;139(1):220–33
